# Silencing of sinusoidal DDR1 reduces murine liver metastasis by colon carcinoma

**DOI:** 10.1038/s41598-020-75395-w

**Published:** 2020-10-27

**Authors:** Irene Romayor, Iker Badiola, Aitor Benedicto, Joana Márquez, Alba Herrero, Beatriz Arteta, Elvira Olaso

**Affiliations:** 1 Tumor Microenvironment Group, Department of Cell Biology and Histology, School of Medicine and Dentistry, University of the Basque Country, 48940 Leioa, Spain; 2 Department of Cell Biology and Histology, School of Medicine and Nursing, University of the Basque Country, 48940 Leioa, Spain

**Keywords:** Experimental models of disease, Liver diseases

## Abstract

Liver metastasis depends on the collagenous microenvironment generated by hepatic sinusoidal cells (SCs). DDR1 is an atypical collagen receptor linked to tumor progression, but whether SCs express DDR1 and its implication in liver metastasis remain unknown. Freshly isolated hepatic stellate cells (HSCs), Kupffer cells (KCs), and liver sinusoidal endothelial cells (LSECs), that conform the SCs, expressed functional DDR1. HSCs expressed the largest amounts. C26 colon carcinoma secretomes increased DDR1 phosphorylation in HSCs and KCs by collagen I. Inhibition of kinase activity by DDR1-IN-1 or mRNA silencing of DDR1 reduced HSCs secretion of MMP2/9 and chemoattractant and proliferative factors for LSECs and C26 cells. DDR1-IN-1 did not modify MMP2/9 in KCs or LSECs secretomes, but decreased the enhancement of C26 migration and proliferation induced by their secretomes. Gene array showed that DDR1 silencing downregulated HSCs genes for collagens, MMPs, interleukins and chemokines. Silencing of DDR1 before tumor inoculation reduced hepatic C26 metastasis in mice. Silenced livers bore less tumor foci than controls. Metastatic foci in DDR1 silenced mice were smaller and contained an altered stroma with fewer SCs, proliferating cells, collagen and MMPs than foci in control mice. In conclusion, hepatic DDR1 promotes C26 liver metastasis and favors the pro-metastatic response of SCs to the tumor.

## Introduction

Collagen deposition promotes tumor development. Up to recently, integrins were considered the unique collagen receptors, but their signaling did not completely explain the underlying mechanism of collagen signaling. Discoidin domain receptor 1 (DDR1) is a tyrosine kinase receptor for collagen that functions as a central sensor of the extracellular matrix (ECM) microenvironment^1,2^. DDR1 is involved in cell adhesion, proliferation, migration and ECM remodeling^2–4^. Together with DDR2, these proteins comprise the family of tyrosine kinase DDRs. DDR1 is an independent prognosis factor for several cancers^5,6^, and is involved in several hallmarks of cancer^7,8^. DDR1 also participates in tissue fibrosis^9,10^ and atherosclerosis^11^. In the last years, intensive research has been carried out to generate new therapies against such severe diseases using DDR1 as a target, some of them are FDA-approved. However, the success of these therapies relates to a more profound knowledge of the mechanism of DDR1 signaling, its cellular localization, and its functional implications in healthy and diseased tissues.

While DDR1 was initially reported in cells of epithelial origin, increasing evidences demonstrate DDR1 expression in cells from the connective, immune, neural and vascular source^12–14^. The use of DDR1KO mice allowed Sun et al. to show that DDR1 drives stromal secretion of IL6 to promote invasive breast cancer^15^, and allowed Hou et al. to demonstrate that DDR1 regulates vascular smooth muscle cell attachment to collagen, chemotaxis, proliferation, and metalloproteinases (MMPs) production^16^. Activation of DDR1 in macrophages stimulates the production of inflammatory cytokines^17^ and nitric oxide, a pivotal modulator of the immune response^18^. While wound healing in DDR1 null mice occurs normally, Chin et al. found that DDR1 plays a crucial role in modulating overexpression of collagen in keloids^19^. Furthermore, Ruiz et al. described that DDR1 drives collagen I-induced human lung fibroblasts migration through collagen IV-coated inserts, but not proliferation^20^. DDR1 deficit in vascular smooth muscle cells leads to mislocalization of adherent contacts^21^. Whether sinusoidal cells (SCs) express DDR1 and its potential implication in liver physiology and pathology remains unknown.

The cellular and molecular composition of the microenvironment is critical for tumor progression^22–24^ and chemoresistance^25^. The liver is the most common site for colorectal carcinoma (CRC) metastasis. Successful arrest and growth of tumor cells occurs within the sinusoidal vessels^26^. The collagen deposition observed afterward induces cancer stiffness that enhances tumor growth and metastatic colonization^27^. Furthermore, experimental models demonstrate that the interplay between collagen, sinusoidal-derived stromal cells, and tumor cells constitute a unique microenvironment that broadly defines the evolution of the metastatic growth^28^. Hepatic stellate cells (HSCs), resident macrophages (Kupffer cells, KCs), and liver sinusoidal endothelial cells (LSECs) are the three major components of the sinusoids. Extensive studies in liver diseases showed that SCs play pivotal roles in hepatic immune, inflammatory, angiogenic, and fibrogenic responses. We have previously demonstrated that SCs exert those functions in experimental liver metastasis^29–32^. Clinically approved multi-kinase inhibitors, such as nilotinib^33,34^ and antibody–drug conjugates^35^ strongly reduced DDR1-mediated CRC cell invasion and metastasis in mouse models, suggesting that DDR1 might be a potential target for cancer treatments. Because nilotinib also affected the host tissue, a role for liver DDR1 could be envisioned. Despite this, nilotinib is not DDR1-specific and, therefore, its effects cannot be ascribed solely to DDR inhibition.

Healthy human liver contains low amounts of DDR1 compared to other organs such as breast^36^ and, thus, hepatic DDR1 effect in liver metastasis has not been sufficiently studied. Intravital microscopy showed that CRC cells mainly arrest in the sinusoidal vessels to initiate their metastatic dissemination and growth, in which extensive collagen deposition composed the associated stroma since very early steps of CRC colonization of the liver. In this work, we utilized a relevant in vitro model of pathological collagen and SCs interaction in the liver and an in vivo model of liver metastases by CRC to study DDR1 expression and its functional consequences in hepatic metastasis. Our previous studies using this model allowed us to focus our research on key aspects of SCs promotion of CRC growth in the liver.

## Results

### DDR1 expression and signaling in hepatic SCs

**Figure 1:**
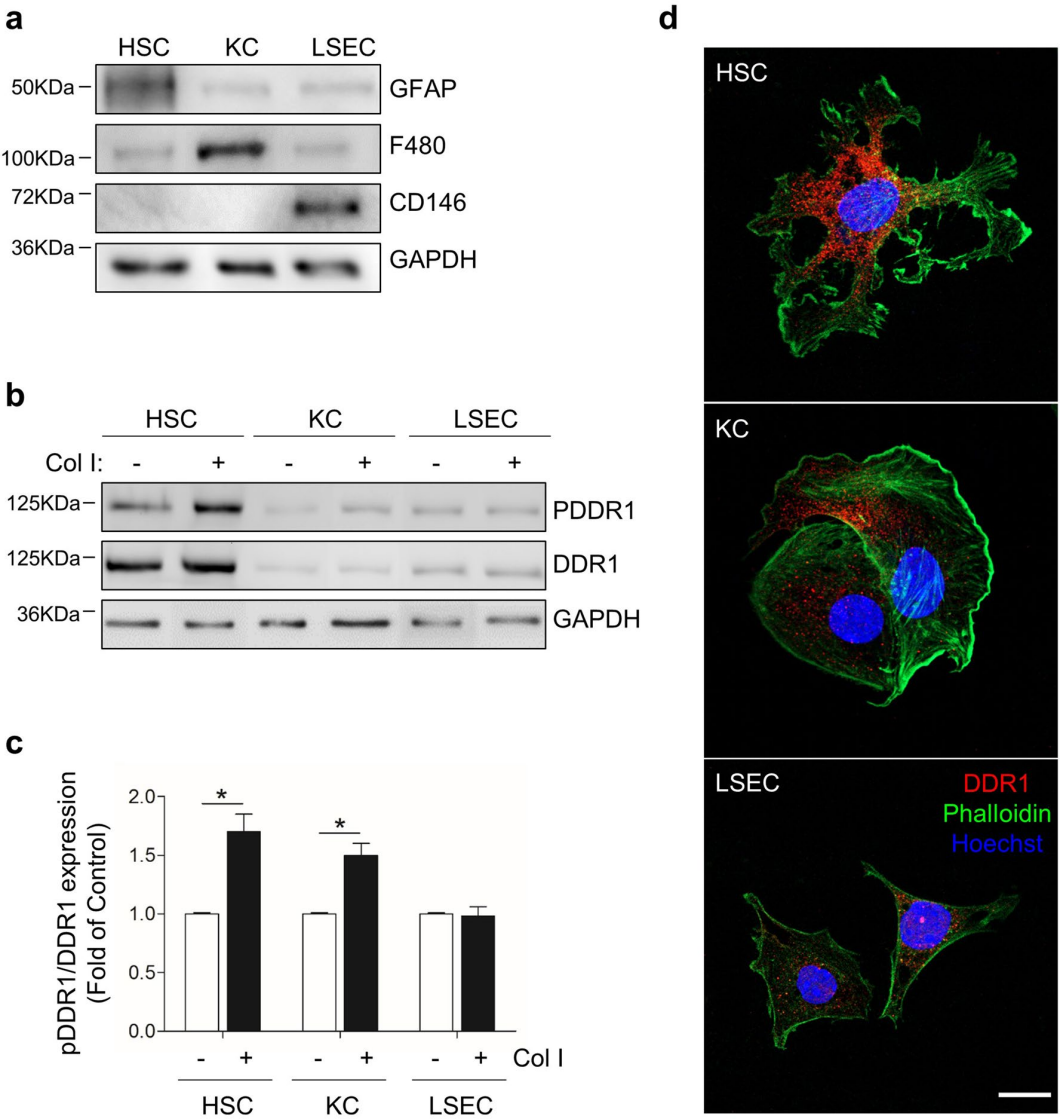
Murine HSCs, KCs and LSECs expression of functional DDR1. SCs were isolated from pooled livers and maintained briefly as monocultures. (**a**) Western blot analysis of the purity of each monoculture using specific cell type markers: GFAP for HSCs, F4/80 for KCs, and CD146 for LSECs. (**b**) Western blot analysis of PDDR1 and total DDR1 expression in the SCs in response to collagen type I. (**c**) Histogram on the semi-quantification of the three experiment performed. (**d**) Immunofluorescence images of DDR1 sub-localization in the SCs. Scale bar 10 µm. Each experiment utilized cells from an independent isolation, and the western blots were processed in parallel. Data are expressed as the means ± SD. *P < 0.05. Full-length blots are presented in Supplementary Figure S8.

To explore DDR1 expression in the murine liver sinusoids, we isolated HSCs, KCs and LSECs, and briefly maintained each of them as monocultures (see Material and Methods). Western blot analysis using cell-type-specific markers indicated a purity of more than ~ 95% for each SC type in the corresponding monoculture (Fig. 1a) (See full-length blots in Supplementary Fig. S8). RT-qPCR demonstrated DDR1 gene expression in all SCs monocultures (see Supplementary Fig. S1).

Western blot analysis showed DDR1 as a band of ~ 125 kDa in lysates from each one of the three SC monocultures. Lysates from human HSCs LX2 and skin fibroblasts derived from DDR1^+/+^ or from DDR1−/− mice were used as positive controls for DDR1 expression (see Supplementary Fig. S2). DDR1 protein expression per cell was ~ fourfold higher in HSCs extracts than in KCs or LSECs ones (Fig. 1b). Exogenous collagen I induced DDR1 phosphorylation by an ~ 1.7 and ~ 1.5-fold increase in HSCs and KCs lysates, respectively, compared to cells in basal media, but it did not increase basal DDR1 phosphorylation in LSECs at the time tested (Fig. 1c). Immunofluorescence staining for DDR1 in the monocultures confirmed these results (Fig. 1d). DDR1 was expressed in the cell membrane and the cytoplasmic compartment of the SCs. Although DDR1 is a membrane receptor, such cellular localization may correspond to protein intracellular trafficking across the cell, as described for DDR1 in renal cells^9^. In consensus with the Western Blot analysis, HSCs displayed the largest amount of DDR1. Similar results were obtained using several anti-DDR1 antibodies (see Supplementary Fig. S2; “Material and methods”). To our knowledge, this is the first study that documents DDR1 in murine liver SCs. It also indicates that the level of DDR1 expression in SCs drastically depends on the cell type. Future analyses will further analyze DDR1 expression and phosphorylation mechanisms in LSECs.

### Increased DDR1 expression by sinusoidal cells in response to C26 cells secretomes

To investigate how DDR1 signaling participates in a pathologic process were collagen plays an important role, we set an in vitro model of tumor-SCs crosstalk. Primary HSCs, KCs, and LSECs monocultures were treated for 1 h with secretomes from C26 cells cultures to obtain tumor-activated SCs or were maintained in basal media. Then, comparative RT-qPCR analyses were performed on a set of pro-inflammatory, immunoregulatory, and pro-fibrogenic genes previously related to liver metastasis by our group^29–32^ (see Supplementary Fig. S1). The gene profile obtained confirmed that C26 cells secretomes generated a pro-metastatic activation program in the SCs. Under those conditions, DDR1 gene expression was enhanced in HSCs and KCs in response to the tumor secretomes, but not in LSECs.

**Figure 2:**
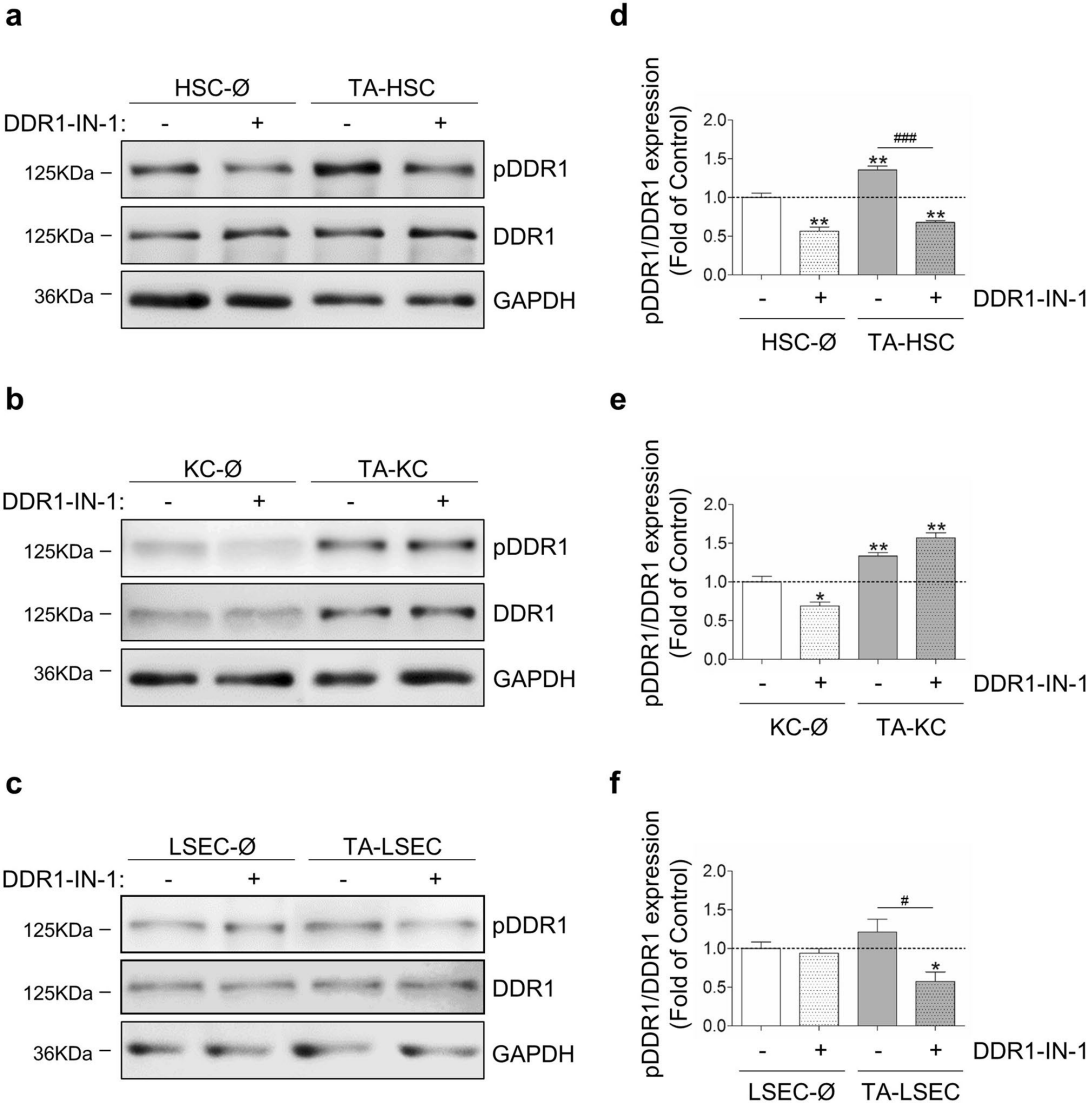
Effect of inhibition of DDR1 kinase activity in the functional response of monocultures of primary SCs to collagen and C26 secretomes. SCs were isolated from pooled livers and maintained briefly as HSCs, KCs and LSECs monocultures. Next, monocultures were maintained under basal conditions (Ø), or in the presence of tumor secretomes to generate tumor activated cells (TA). Some cells were maintained for 2 h in the presence of collagen type I with or without 70 nM DDR1-IN-1. Lysates were analyzed by Western blot. (**a**–**c**) Western blot analyses of PDDR1 and total DDR1 expression in HSCs (**a**), KCs (**b**) and LSECs (**c**). (**d**,**e**) Histograms on computer-assisted semi-quantification of the three experiments performed in HSCs (**d**), KCs (**e**) and LSECs (**f**). Each experiment utilized cells from an independent isolation, and the western blots were processed in parallel. Data are expressed as the means ± SD. */^#^P < 0.1, **P < 0.01, ^###^P < 0.001. Full-length blots are presented in Supplementary Figure S8.

Next, we compared DDR1 protein expression and phosphorylation between SCs maintained overnight in either C26 cells secretomes (tumor-activated SCs) or basal media (Fig. 2). Tumor secretomes enhanced DDR1 protein expression by ~ 1.4 fold in KCs (Fig. 2b), while it did not modify DDR1 protein levels in HSCs (Fig. 2a) or LSECs (Fig. 2c). Tumor secretomes promoted DDR1 phosphorylation by collagen I in HSCs by ~ 1.5 fold and in KCs by ~ 1.4 fold, but not in LSECs, under the conditions used (see full-length blots in Supplementary Fig. S8). To explore the role of DDR1 signaling in SCs, we used DDR1-IN-1, a type II kinase inhibitor with high specificity for DDR1^37^. Although DDR1-IN-1 can target DDR2 (IC_50_ = 413 nM), its inhibitory effect is fourfold less potent compare to DDR1 (IC_50_ = 105 nM)^38^. To set the appropriated concentration and time conditions for DDR1-IN-1 treatment of SCs, we utilized two cell lines: 3T3 fibroblasts and J774A.1 macrophages, known to express a DDR1 that strongly responses to collagen I (see Supplementary Fig. S3). The two cell lines were cultured in the presence of increasing concentrations of DDR1-IN-1 (see Supplementary Figs. S4, S5). The results indicated that 1-h pretreatment with DDR1-IN-1 followed by 2-h treatment with collagen I and DDR1-IN-1 results in a 50% decrease of DDR1 phosphorylation at a 70–100 nM concentration range. A 70–100 nM DDR1-IN-1 for a total of 3 h was tested in SCs. 100 nM promote stress fiber formation in HSCs. Longer culture time and/or higher DDR1-IN-1 concentrations promoted KCs and LSECs detachment, and thus, results were not considered. 70 nM was finally selected for further experiments because it did not promote cell detachment or stress fiber formation. More importantly, 70 nM DDR1-IN-1did not promote apoptosis in the SCs, as demonstrated by the lack of active Caspase 3 in the cell cultures (see Supplementary Fig. S6). DDR1-IN-1 decreased DDR1 phosphorylation by collagen I in basal HSCs by ~ 50%. Even more, DDR1-IN-1 lowered DDR1 phosphorylation in tumor-activated HSCs by ~ 40% (Fig. 2). DDR1-IN-1 reduced PDDR1 in basal KCs by ~ 30%, but was unable to inhibit PDDR1 in tumor-activated KCs under the conditions used. Finally, DDR1-IN-1 did not significantly modified PDDR1 levels in LSECs (Fig. 2d–f).

### Inhibition of DDR1 kinase activity modifies key functional prometastatic responses of primary sinusoidal cells to C26 cells secretomes in the presence of collagen I

**Figure 3:**
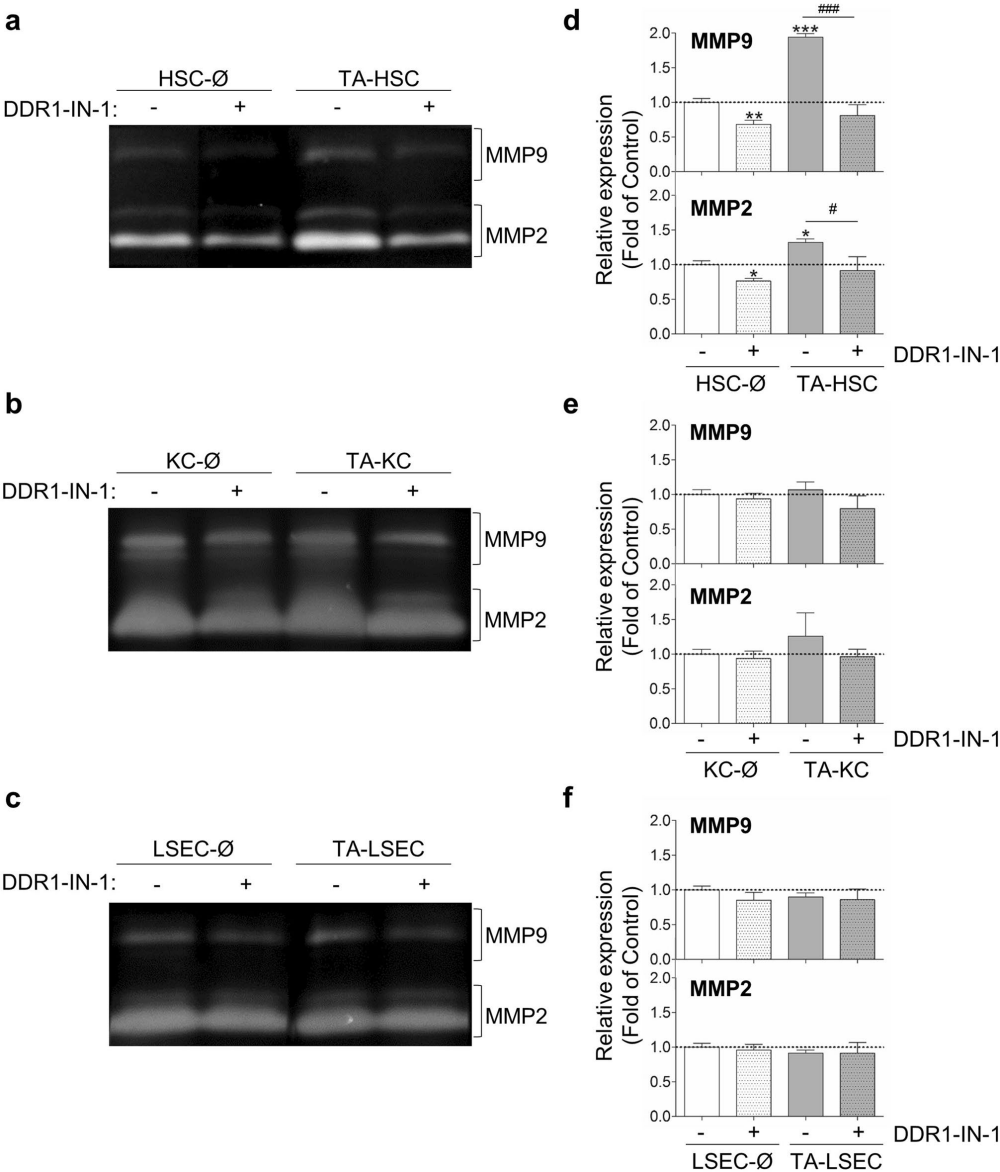
DDR1 kinase‐dependent expression of MMPs by SCs. Primary monocultures of basal (Ø) or tumor-activated (TA) HSCs, KCs and LSECs were pretreated or not with DDR1-IN-1 and collagen I. Then media was changed to fresh basal media, and secretomes collected 10 h afterwards, and submitted to gelatin zymography. (**a**–**c**) Gelatin zymography of HSCs (**a**), KCs (**b**) and LSECs (**c**) secretomes. (**d**–**f**) Histograms on computer-assisted semi-quantitation of the three experiment performed in HSCs (**d**), KCs (**e**) and LSECs (**f**). Data represents the total amount of each MMP, expressed as the sum of the intensity of the bands corresponding to proMMP and active MMP in SCs secretomes. Each experiment utilized cells from an independent isolation, and the zymographies were processed in parallel. Data are expressed as the means ± SD. */^#^P < 0.01, ***/^###^P < 0.001. Histogram on MMP9 expression in KCs is presented in Supplementary Figure S7.

Next, we utilized DDR1-IN-1 to analyze if DDR1 phosphorylation mediates SCs pro-metastatic behavior. We focused on two sinusoidal cell functions essential for tumor growth: regulation of MMPs synthesis and secretion of proliferative and migratory factors. Basal-SCs or tumor activated-SCs monocultures were treated or not with DDR1-IN-1 and collagen I (see “Material and methods”). The amounts of gelatinases (MMP2 and MMP9) in the resulting secretomes were analyzed by gelatin zymography (Fig. 3). A mixture of recombinant MMP2/MMP9 was used as control to better address the nature of each band (data not shown). Each of the MMPs appears as bands of different sizes, according to their processing status, the major bands being ~ 92KDa (pro-MMP9), ~ 82KDa (active MMP9), ~ 72 kDa (pro-MMP2) and ~ 62 kDa (active MMP2). The three SCs monocultures secreted MMPs to the culture media. Tumor activation induced a significant increase in MMPs secretion only in HSCs, under the conditions used. DDR1-IN-1 decreased total MMP2 and total MMP9 (mainly detected as pro-MMPs and active MMPs) secretion by basal HSCs and tumor-activated-HSCs (Fig. 3a,d). These results correlate with the inhibition of PDDR1 by DDR1-IN-1 in HSCs (Fig. 2). On the other hand, total MMPs secretion was not modified in KCs neither by tumor secretomes nor by DDR1-IN-1, compared to that of untreated basal KCs (Fig. 3b). Despite this, analysis of each MMP band in each of the three independent experiments showed that DDR1-IN-1 significantly reduced by ~ 25% the expression of the active MMP9 (~ 82KDa) in the secretomes from basal KCs cultures, and by ~ 50% in the secretomes from tumor-activated KCs (see Supplementary Fig. S7). MMP2 was ~ 20% lower in KCs treated with DDR1-IN-1, but it was not statistically significant. Finally, there were no statistically significant changes in MMPs secretion by LSECs under any of the assayed conditions (Fig. 3c,f).

Next, the SC secretomes were utilized as a source of proliferative or chemotactic factors for primary LSECs or C26 cells. Some control C26 cells were cultured in basal media, while the remaining were cultured in SCs secretomes. C26 cells proliferation was measured afterward. Other C26 cells were seeded in a collagen-coated upper well of a modified Boyden chamber to analyze their invasion rate in response to the chemotactic effect of control conditions or SCs secretomes located in the lower chamber.

**Figure 4:**
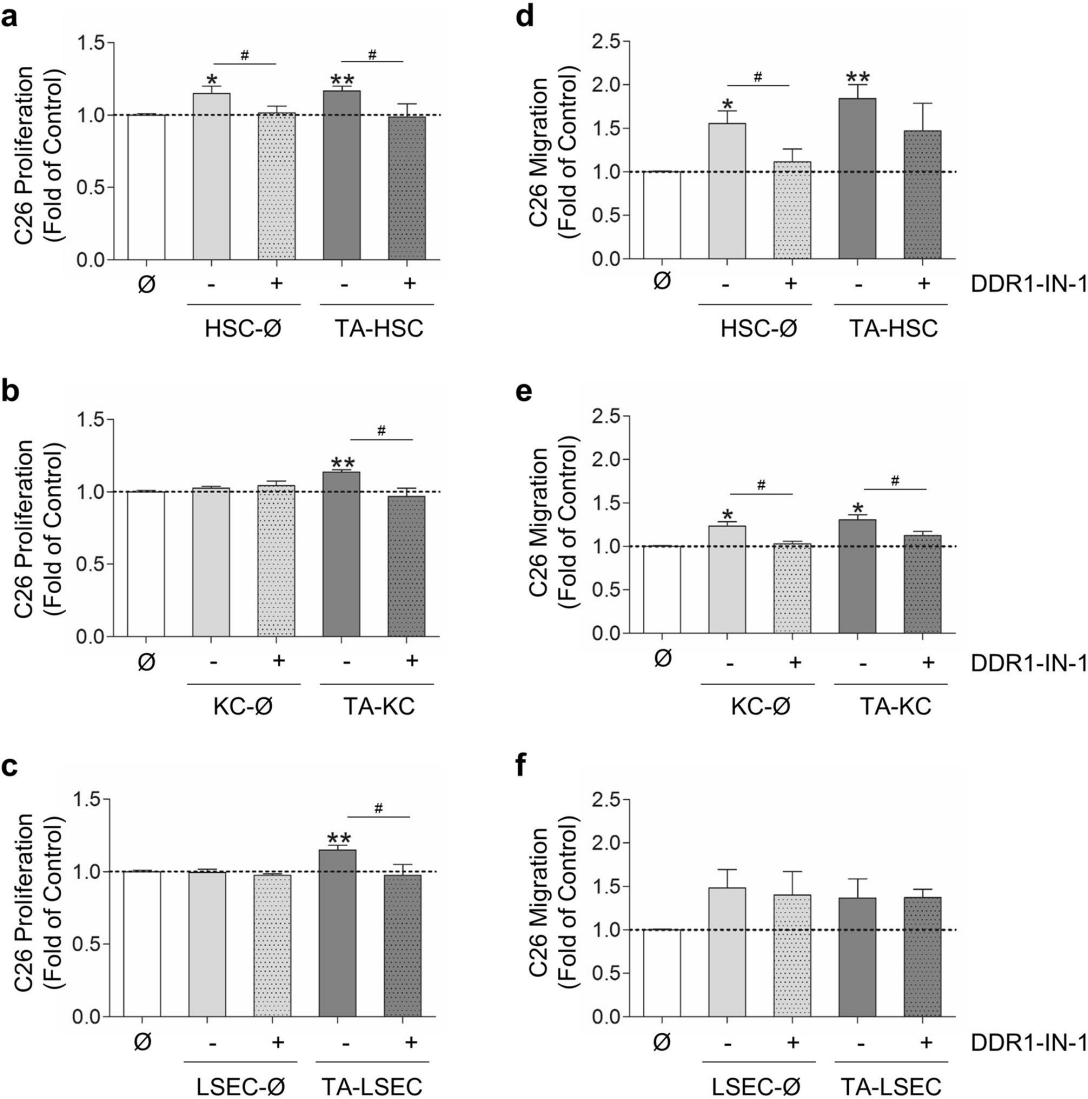
DDR1 kinase‐dependent secretion of chemotactic and proliferative factors for C26 cells by SCs. Primary monocultures of basal (Ø) or tumor-activated (TA) HSCs, KCs and LSECs were pretreated or not with DDR1-IN-1 and collagen I. Then media was changed to fresh basal media, and secretomes collected 10 h afterwards. Those secretomes were utilized as a source of proliferative or chemotactic factors for C26 cells. Control C26 cells were cultured in basal media, while the remaining C26 were cultured in SCs secretomes. C26 cells proliferation was measured afterward. Other C26 cells were seeded in a collagen-coated upper well of a modified Boyden chamber to analyze their invasion rate in response to the chemotactic effect of SCs secretomes, located in the lower chamber. Control cells were allowed to migrate in response to basal media. (**a**–**c**) Tumor proliferation in response to secretomes from HSCs (**a**), KCs (**b**) and LSECs (**c**). (**d**–**f**) Tumor migratory activity in response to secretomes from HSCs (**d**), KCs (**e**) and LSECs (**f**). Each experiment utilized cells from an independent isolation. Data are expressed as means ± SD.*/^#^P < 0.1, **P < 0.01.

Secretomes from basal and tumor-activated HSCs promoted C26 cells proliferation and migration. DDR1 blockage decreased both C26 growth and C26 migration to control levels, in response to basal HSCs secretomes. On the other hand, DDR1-IN-1 reduced the induction of C26 proliferation by tumor-activated HSCs, but it did not affect significantly C26 migration (Fig. 4a,d).

Tumor-activated KCs secretomes, but no basal KCs secretomes, enhanced C26 proliferation rate, which was reverted by DDR1-IN-1 (Fig. 4b). Secretomes from basal and tumor-activated KCs also contained chemotactic factors for C26 cells, whose secretion was mainly DDR1-kinase-dependent (Fig. 4e). Finally, secretomes from tumor-activated LSECs, but no basal LSECs secretomes, increased tumor proliferation. In this case, DDR1-IN-1 completely blocked the proliferative effect (Fig. 4c). LSECs-derived factors did not affect significantly tumor migration (Fig. 4f).

These results indicate that DDR1 phosphorylation in HSCs supports the most robust modulation of cell secretomes among SCs to promote MMPs secretion, tumor proliferation and chemotactic migration. We next utilize mRNA silencing as a second approach to block DDR1. We focused exclusively on DDR1 in HSCs because our results indicated that these cells are the main source of DDR1 in the sinusoids, and showed the more robust changes under DDR1-IN-1 treatment. We also selected HSCs because, as opposed to KCs and LSECs, culture on plastic surface prompt quiescent HSCs to transdifferentiate from vitamin A-rich cells to the myofibroblast-like ones present inside liver metastases^31,32^.

### DDR1-dependent genes in HSCs

**Figure 5:**
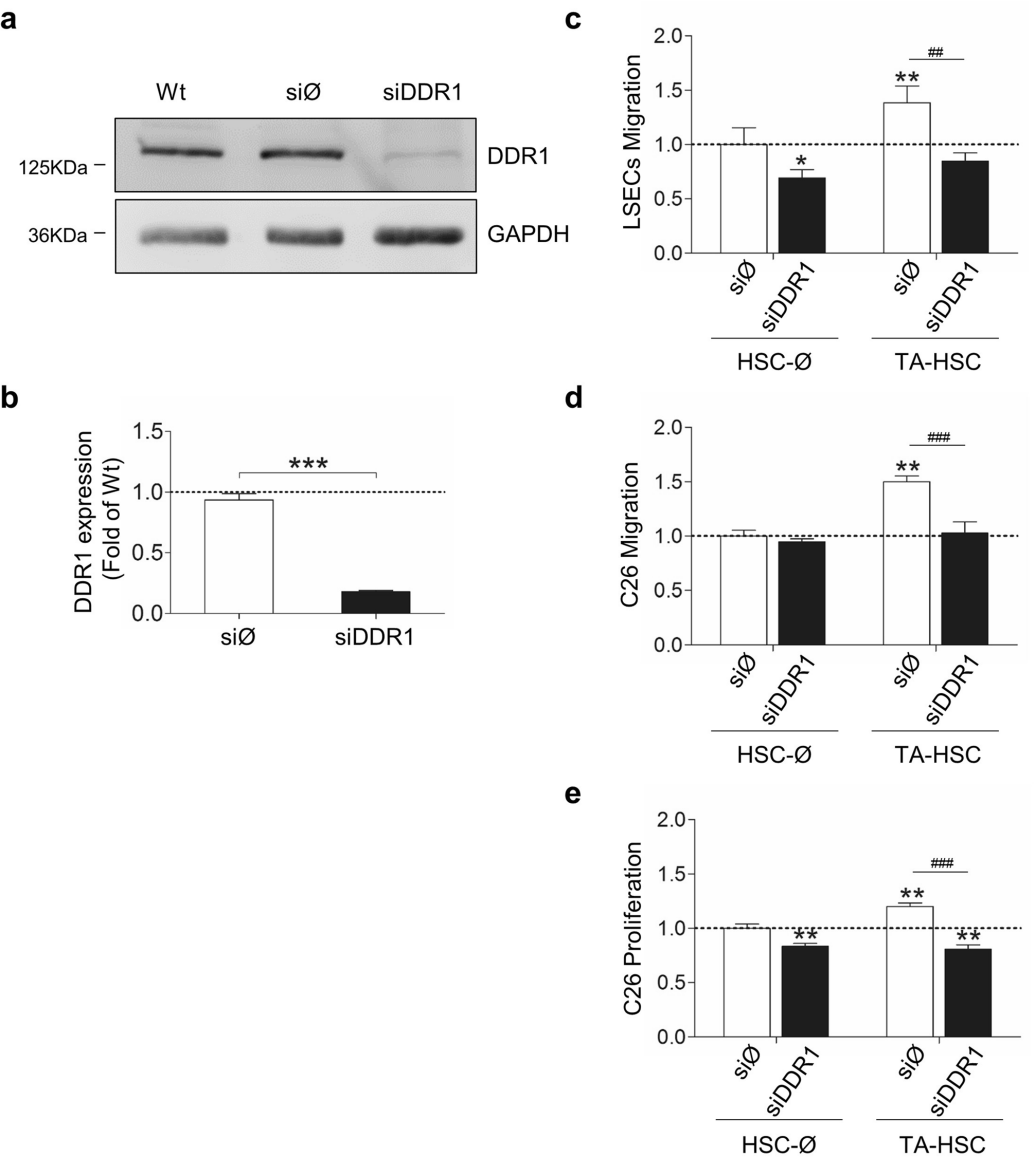
Silencing of DDR1 reduces HSCs secretion of chemotactic factors for both LSECs and C26 cells, and proliferative factors for C26 cells. (**a**) Western blot analysis of DDR1 in HSCs silenced (siDDR1) or not (siØ) for DDR1mRNA. (**b**) Histograms on computer-assisted semi-quantification of the Western Blot. LSECs or C26 cells were seeded in a collagen-coated upper well of a modified Boyden chamber to analyze their invasion rate in response to the chemotactic effect of SC secretomes located in the lower chamber. Control cells were allowed to migrate in response to basal media. (**c**,**d**) Chemotactic migration of LSECs (**c**) or C26 cells (**d**) in response to HSCs secretomes. (**e**) Proliferation rate of C26 cells in response to HSCs secretomes. Data are expressed as means ± SD. *P < 0.1, **/^##^P < 0.01, ^###^P < 0.001. Each experiment utilized cells from an independent isolation, and the western blots were processed in parallel. Full-length blots are presented in Supplementary Figure S8.

HSCs monocultures were maintained in culture for 2 days and then transfected with either an irrelevant si mRNA (siØ), a siDDR1, or remained untreated (see Material and Methods). Then, media was changed and subconfluent HSCs remained untreated for 3 days to allow maximal inhibition of DDR1 expression. Since day three of culture, endogenous collagen was enough to maintain DDR1 basally phosphorylated (see Fig. 1, lane 1 from the left). At day five after transfection, Western blot showed that siDDR1 HSCs expressed ~ 80% less DDR1 than HSCs siØ or untransfected HSCs (Fig. 5a,b). At that time point, total RNAs were isolated and hybridized onto DNA microarrays for gene expression analysis. According to the hybridization, 327 genes were twofold or more upregulated in the siDDR1 HSCs compared with siØ cells, while 976 genes were twofold or more downregulated. Gene ontology (GO) was carried out in both sets of genes (Tables 1, 2). The genes which were up- or down-regulated in the siDDR1 HSCs were introduced into the AmiGO 2 database.

**Table 1:** Genes down-regulated in DDR1 silenced Hepatic Stellate Cells.

PANTHER GO-slim biological process	Upload_Genes (976)	(P value)
Granulocyte chemotaxis (GO:0071621)	19	8.79E−09
Leukocyte migration (GO:0050900)	22	1.53E−09
Leukocyte chemotaxis (GO:0030595)	19	6.89E−08
Positive regulation of GTPase activity (GO:0043547)	9	1.48E−02
Inflammatory response (GO:0006954)	33	7.79E−14
Defense response to virus (GO:0051607)	10	1.26E−02
Cellular response to tumor necrosis factor (GO:0071356)	9	3.81E−02
Positive regulation of ERK1 and ERK2 cascade (GO:0070374)	10	1.58E−02
Cellular response to cytokine stimulus (GO:0071345)	26	7.94E−08
Response to cytokine (GO:0034097)	28	5.32E−08
Regulation of hydrolase activity (GO:0051336)	17	6.66E−04
Cytokine-mediated signaling pathway (GO:0019221)	21	3.78E−05
Cell migration (GO:0016477)	35	9.21E−10
Response to lipopolysaccharide (GO:0032496)	16	2.88E−03
Cellular response to lipopolysaccharide (GO:0071222)	14	1.41E−02
Localization of cell (GO:0051674)	35	5.01E−08
Cell motility (GO:0048870)	35	5.01E−08
Response to organic substance (GO:0010033)	40	6.38E−06
Divalent inorganic cation homeostasis (GO:0072507)	22	3.94E−02
Regulation of catalytic activity (GO:0050790)	26	7.82E−03
Defense response (GO:0006952)	47	3.50E−06
MAPK cascade (GO:0000165)	27	1.61E−02
Apoptotic process (GO:0006915)	33	5.74E−03
Cell adhesion (GO:0007155)	42	4.06E−04
Biological adhesion (GO:0022610)	42	4.06E−04
Signal transduction by protein phosphorylation (GO:0023014)	28	4.58E−02
Programmed cell death (GO:0012501)	33	9.07E−03
Regulation of molecular function (GO:0065009)	38	3.93E−03
Protein phosphorylation (GO:0006468)	43	8.99E−04
Cell death (GO:0008219)	33	2.62E−02
Response to stress (GO:0006950)	53	1.95E−04
Immune system process (GO:0002376)	88	5.44E−08
Response to chemical (GO:0042221)	61	2.43E−04
Response to stimulus (GO:0050896)	125	5.10E−11
Cellular protein metabolic process (GO:0044267)	83	1.40E−03
Cellular macromolecule metabolic process (GO:0044260)	83	1.47E−03
Intracellular signal transduction (GO:0035556)	63	4.87E−02
Signal transduction (GO:0007165)	127	3.92E−04
Cellular response to stimulus (GO:0051716)	139	1.63E−03

Genes down-regulated in DDR1 silenced hepatic stellate cell according to the gene array were introduced into the AmiGO 2 Gen onthology database by using Biological Process analysis. The table shows the gene number into each Go-slim Biological process Bonferroni-corrected for P < 0.05.

**Table 2:** Genes upregulated in DDR1 silenced hepatic stellate cells.

PANTHER GO-Slim Biological Process	Upload_genes (327)	(P-value)
Vasculature development (GO:0001944)	24	3.82E−02
Regulation of apoptotic process (GO:0042981)	48	5.48E−03
Regulation of programmed cell death (GO:0043067)	48	7.03E−03
Regulation of cell death (GO:0010941)	52	3.99E−03
Regulation of cell population proliferation (GO:0042127)	51	6.62E−03
Animal organ development (GO:0048513)	84	7.92E−05
Positive regulation of nitrogen compound metabolic process (GO:0051173)	83	2.85E−04
Regulation of signaling (GO:0023051)	85	8.01E−04
positive regulation of macromolecule metabolic process (GO:0010604)	85	8.40E−04
Negative regulation of cellular process (GO:0048523)	121	2.22E−07
Regulation of developmental process (GO:0050793)	70	1.85E−02
Regulation of signal transduction (GO:0009966)	73	1.21E−02
Regulation of cell communication (GO:0010646)	84	1.19E−03
Positive regulation of cellular metabolic process (GO:0031325)	85	9.19E−04
Regulation of biological quality (GO:0065008)	101	8.42E−05
Positive regulation of metabolic process (GO:0009893)	90	1.10E−03
Positive regulation of cellular process (GO:0048522)	137	4.63E−08
Negative regulation of biological process (GO:0048519)	130	3.38E−07
Regulation of multicellular organismal process (GO:0051239)	81	1.00E−02
System development (GO:0048731)	104	2.89E−04
Positive regulation of biological process (GO:0048518)	148	7.92E−08
Cell differentiation (GO:0030154)	88	2.66E−02
Cellular developmental process (GO:0048869)	89	3.38E−02
Developmental process (GO:0032502)	129	9.99E−05
Anatomical structure development (GO:0048856)	120	6.88E−04
Multicellular organism development (GO:0007275)	110	6.17E−03
Regulation of cellular metabolic process (GO:0031323)	123	2.77E−03
Regulation of nitrogen compound metabolic process (GO:0051171)	115	1.14E−02
Regulation of macromolecule metabolic process (GO:0060255)	121	4.63E−03
Regulation of metabolic process (GO:0019222)	131	1.79E−03
Regulation of primary metabolic process (GO:0080090)	117	1.48E−02
Biological regulation (GO:0065007)	240	2.04E−08
Regulation of cellular process (GO:0050794)	213	1.65E−05
Response to stimulus (GO:0050896)	166	2.02E−02
Regulation of biological process (GO:0050789)	226	1.80E−06
Cellular process (GO:0009987)	263	1.58E−07
Unclassified (UNCLASSIFIED)	9	0.00E00

Genes up-regulated in DDR1 silenced hepatic stellate cell according to the gene array were introduced into the AmiGO 2 Gen onthology database by using biological process analysis. The table shows the gene number into each Go-slim Biological process Bonferroni-corrected for P < 0.05.

Table 3 shows selected genes differentially expressed between siDDR1 silenced and non-silenced HSCs that had been previously reported to be altered in SCs during liver metastases, arranged by their inclusion in GO terms. The silencing of DDR1 in HSCs reduced the expression of genes for collagen II, III and IV, and collagen catabolism and remodeling, including MMPs. A second large set of downregulated genes belonged to the inflammatory response, including genes for interleukins IL1, IL6, IL13 and IL18. Genes related to cell migration, such as those of the CXCL family, were also downregulated. Several genes that regulate cell proliferation and HSCs transdifferentiation, such as those for PDGF receptors, FAP, STAT1 and CSF1-2, were less expressed in the silenced cells. Finally, genes related to cell adhesion such as the ECM components tenascin, laminin, versican and dystroglycan were also downregulated. Several upregulated genes were related to cell cycle arrest, such as p53-dependent cyclins and regulation of protein phosphorylation such as AKT.

**Table 3:** Gene Ontology (GO) analysis on selected HSCs genes modified by DDR1 mRNA silencing.

	Downregulated	*P* value
Collagen fibril organization	Col2α1, Col3α1,Col14α1, Loxl3	1.23E−06
Collagen catabolic process	Mmp3, Mmp9, Mmp12, Mmp13	4.96E−08
Inflammatory response	Il1a, Il1b, Il6, Il13, Il18, Il1rn, Il1rl2	3.02E−05
Inflammatory response	Cxcl1, Cxcl2, Cxcl5, Cxcl9, Cxcl10, Cxcl11, Cxcl12, Cxcl13	7.81E−08
Regulation of inflammatory response	Tnf , Tnfrsf11a, Tnfsf4, Tnfaip3, Tnfaip6	3.22E−04
Chemokine-mediated signaling pathway	Cxcl1, Cxcl2, Cxcl5, Cxcl9, Cxcl10, Cxcl11, Cxcl12, Cxcl13	2.53E−17
Regulation of cell proliferation	Pdgfra, Pdgfrb, Pdgfc, Fap, Csf1, Csf2, Stat1, Fgfr1op	7.67E−04
Cell adhesion	Tnc, Lamb1-1, Vcan, Lama4, Dag1, Hpse	5.54E−03
Protein autoprocessing	Casp1, Casp 12	4.74E−03

	Upregulated	*P* value
Mitotic cell cycle	Cdkn1a, Ccno, Ccng1, Hus1, Gtse1, Cep55, Ckap2	3.14E−02
Regulation of cyclin-dependent protein serine/threonine kinase activity	Cdkn1a, Akt1, Ccno, Ccng1	2.84E−02
Regulation of protein phosphorylation	Cdkn1a, Akt1, Ccno, Ccng1, Hus1, Cblb, Gdf15, Ccl19, Ifna2	4.42E−02

Genes were introduced into the AmiGO 2 Gen Ontology database, biological process. Bonferroni-corrected for P < 0.05.

### Silencing of DDR1 reduces the ability of tumor-activated HSCs to promote migration of LSECs and C26 cells across collagen I and tumor proliferation

Accumulating in vivo and in vitro data suggest that activated HSCs promote tumor cell migration, survival, and growth and mediates the angiogenic migration of LSECs into the developing tumor along the metastasis process^31,32,39^. Because the gene array favors the notion that HSCs secretion of chemotactic factors is DDR1-dependent, primary LSECs and C26 cells were cultured in a modified Boyden chamber and allowed to migrate across collagen I (Fig. 5c,d). Secretomes from HSCs, untransfected or transfected with either siØ or siDDR1, and maintained under basal conditions or activated by pretreatment with C26 secretomes (TA) were used as chemoattractants. Secretomes from siØ HSCs under basal conditions did not affect endothelial or tumor migration significantly, while secretomes from siØ-TA HSCs induced endothelial migration by ~ 1.25 fold, and tumor cells by ~ 1.5 fold. SiDDR1-TA HSCs generated secretomes that did not enhance endothelial and tumor cells migration above the levels generated by secretomes from siDDR1-basal HSCs. Finally, soluble factors from siDDR1-TA HSCs reduced tumor proliferation below that generated by siØ-basal HSCs (Fig. 5e) (see full-length blot in Supplementary Fig. S8). There were no significant differences between the effect generated by secretomes from untreated and siØ HSCs in any experiments performed with LSECs or C26 cells.

As a whole, these data show that tumor cells exposed to secretomes from DDR1 silenced HSCs showed a less aggressive invasive behavior than their counterparts exposed to DDR1-expressing HSCs. Moreover, DDR1 silencing also decreased the pro-angiogenic phenotype of TA-HSCs.

### In vivo silencing of mouse DDR1 expression reduces liver metastasis

**Figure 6:**
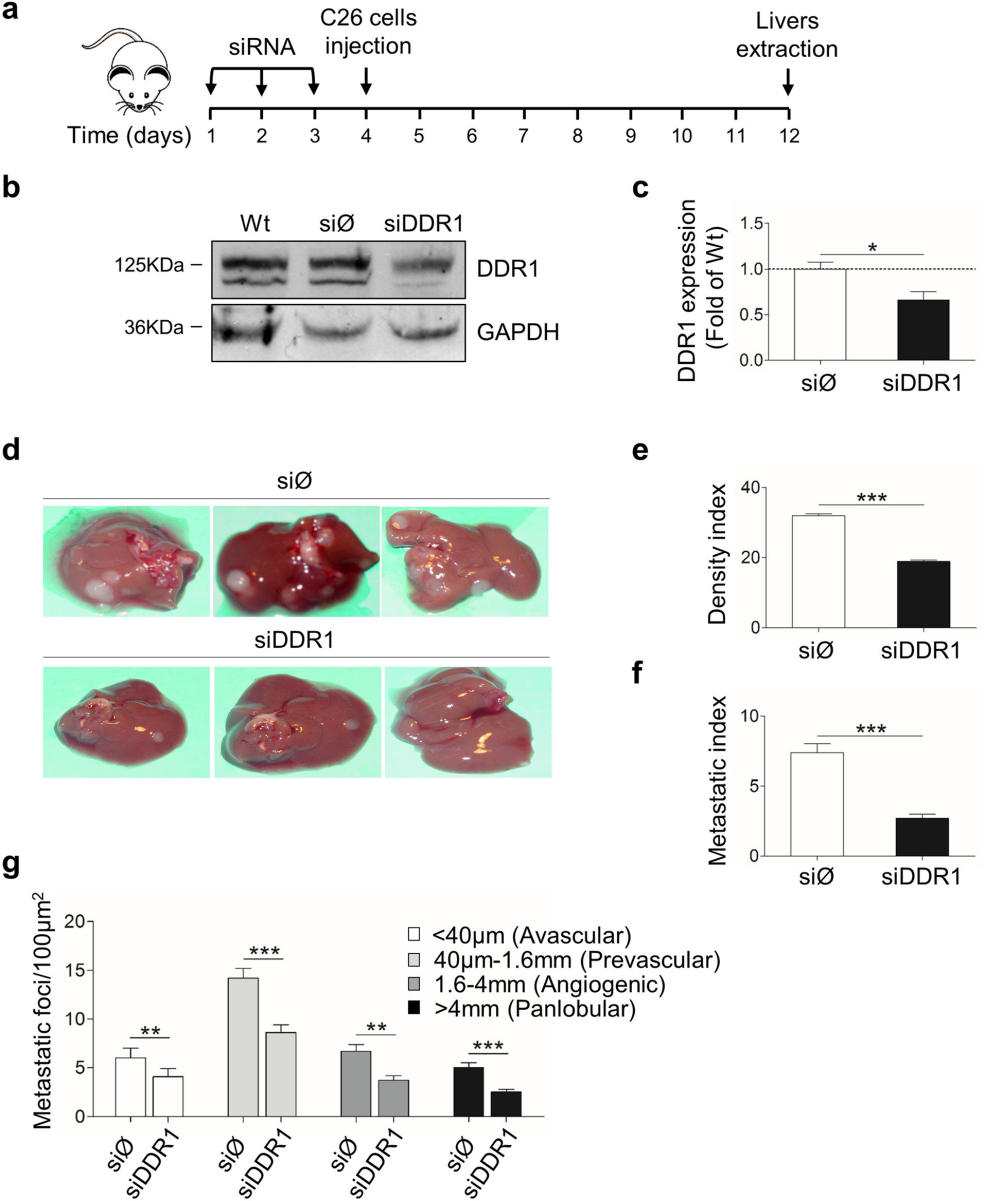
Silencing of hepatic DDR1 reduces experimental liver metastasis by C26 cells. (**a**) Treatment schedule. (**b**) Western Blot of DDR1 expression in liver lysates from tumor-free mice injected with the silencing or control RNAs at day 8 post-treatment. (**c**) Histograms on computer-assisted semi-quantitation of western blots for DDR1 expression in siDDR1 and siØ livers. (**d**) Representative murine livers 8 days after tumor inoculation to the spleen. (**e**) Number of metastatic foci per liver area. (**f**) Percentage of total liver volume occupied by metastasis. (**g**) Distribution of metastatic foci according to their developmental stage. Histograms in (**e**–**g**) represent averaged values of 20 micrometastatic foci per mice, in 24 mice with liver metastases. Data are presented as means ± SD. *P < 0.1, **P < 0.01, ***P < 0.001. The experiment was repeated twice. Full-length blot is presented in Supplementary Figure S8.

To analyze the role of DDR1 in the early steps of liver metastases, we transiently silenced hepatic DDR1 by three consecutive injections of siDDR1 RNA. A siØ group of mice was injected with an irrelevant siRNA, and a third group of mice remained untreated. Then, tumor cells were injected in the spleen of mice untreated or treated with either siDDR1 or siØ 24 h after the last siRNA injection (Fig. 6a). Western Blot analysis of total liver lysates at day 8 of treatment showed that DDR was reduced in siDDR1 livers by a ~ 65% (Fig. 6b–d). All mice presented liver metastases 10 days after tumor injection. As described previously^40^, the main mechanism of hepatic C26 metastasis growth was of the hepatic tissue replacement-type, characterized by an efficient invasive behavior of cancer cells. Also, cell of the tumor microenvironment conformed predominantly a sinusoidal type, containing a rich network of intratumoral microvessels supported by sinusoidal-derived myofibroblasts. Histochemical analysis showed a ~ 40% reduction in the volume occupied by metastasis and a ~ 27% decrement in the density of metastatic foci in siDDR1 mice, compared to siØ or untreated mice (Fig. 6e,f). No significant differences were observed between tumor development in siØ and untreated mice.

We arbitrarily divided all hepatic micrometastases in four groups, according to their size and developmental stage (Fig. 6g). Silencing of hepatic DDR1 resulted in a ~ 28% reduction in the number of small avascular micrometastases characterized by their lack of infiltrating stroma, which may indicate a role for hepatic DDR1 in tumor cell implantation. The majority of hepatic micrometastases were at a prevascular/angiogenic stage of growth, characterized by the highest densities of angiogenic and desmoplastic stromal reaction. They composed a ~ 70% of tumor foci per observed area in siØ mice and a ~ 60% in siDDR1 ones. A comparison between those groups showed a ~ 44% reduction in tumor density in siDDR1. Finally, panlobular metastases were ~ 58% lower in the siDDR1 livers. Central necrosis was rarely observed in the tumor foci under any treatment at this stage of development. As a whole, these results may indicate that DDR1-silenced livers offer less favorable microenvironmental conditions for both implantation and growth of CRC metastasis in mice.

**Figure 7:**
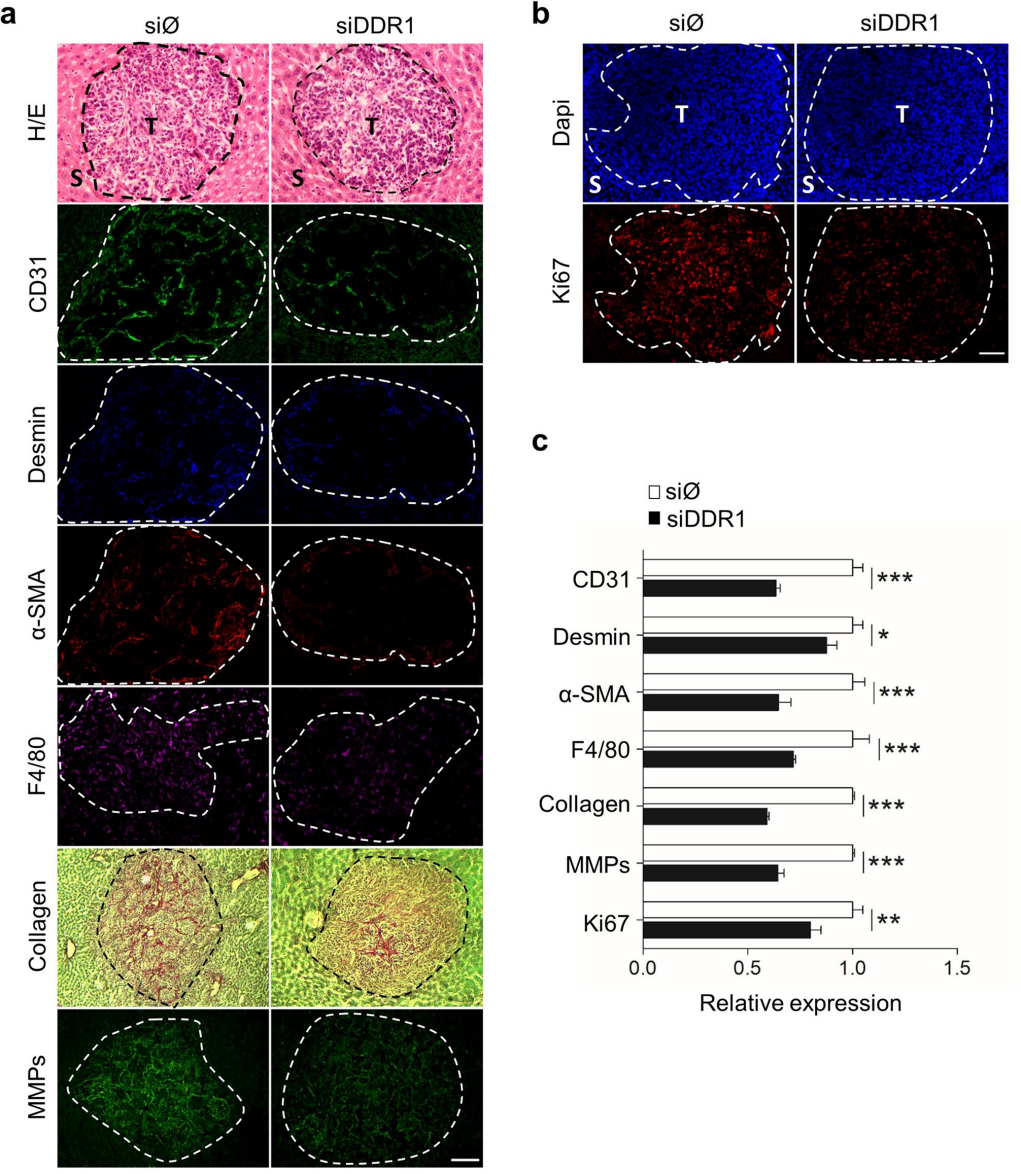
Immunohistochemical analysis of experimental hepatic metastasis by C26 cells in mice pretreated or not with DDR1 silencing RNA. (**a**) Immunofluorescence images of intratumoral expression of CD31/desmin/α-SMA in triple-labeled liver sections, and F4/80, Picrosirius Red, and in situ zymography in serial liver sections. (**b**) Representative images of DAPI and Ki67 staining in serial liver tissue sections. Liver tissue sections were obtained from the mouse analyzed in Fig. 6. *T* tumor, *S* sinusoids. Dotted white lines separate the tumor from the surrounding hepatic tissue. Scale bar 50 µm. (**c**) Histogram on computer-assisted semi-quantitation represents averaged values of 20 tumor foci per mice, in 24 mice with liver metastases. Data are expressed as means ± SD *P < 0.1, **P < 0.01, ***P < 0.001. The experiment was repeated twice. All images were processes under the same conditions.

Next, we investigated whether the composition of the tumor stroma is affected by the level of hepatic DDR1. Tumor foci at the prevascular/angiogenic stages of metastatic growth were analyzed by immunofluorescence and confocal microscopy (Fig. 7). Analysis of triple stained liver sections revealed that silencing of hepatic DDR1 reduced the intratumoral density of angiogenic, CD31-expressing LSECs by ~ 40%, desmin expressing HSCs by ~ 10% and α-SMA expressing myofibroblast by ~ 37%. 85% of α-SMA-expressing cells also contained desmin, indicating an HSC origin. Immunofluorescence on serial sections also revealed a ~ 30% reduction in the density of recruited F4/80-expressing KCs. Furthermore, Picrosirius staining of collagen was ~ 42% lower in siDDR1 than in siØ livers, and correlated with a 40% reduction in the in situ MMPs activity (Fig. 7a,c). Finally, a ~ 18% decrement was observed in the density of Ki67-expressing proliferating cells in siDDR1 hepatic tissue, compared to the control (Fig. 7b,c). No statistical differences existed between untreated and siØ mice. Collectively, the data indicate that the silencing of hepatic DDR1 impairs the generation of a pro-angiogenic, pro-fibrogenic and proliferative microenvironment provided by the crosstalk between tumor cells and the surrounding SCs.

## Discussion

Although DDR1 is mostly expressed by epithelial cells, previous studies indicated that non-epithelial cells, such as myofibroblast-like cells in cancerous tissues, also express DDR1^41^. In this study, we demonstrated for the first time that freshly isolated HSCs, KCs and LSECs of the murine liver capillaries express DDR1. While no report exists on DDR1 in murine liver, expression of DDR1 has been described in human hepatocytes and cholangiocytes by immunohistochemistry analyses of human liver sections^9,42^. Interestingly, none of the reports utilized SCs markers nor studied DDR1 expression in isolate liver cell cultures. In this regard, we have reported robust DDR1 expression in the human HSCs line LX2^43^.

The dysregulation of matricellular components of the tumor microenvironment has been linked with the development of metastases in multiple cancer types^24^. Increased production of collagen in and around hepatic metastases occurs in humans^27^, but its clinical implications are still not well understood. Experimental models have demonstrated that the crosstalk between metastatic CRC cells and the hepatic sinusoidal occurs in a collagenous microenvironment since very early stages of tumor growth. To this regard, we found that DDR1mRNA expression in SCs increases in response to tumor secretomes at a time when gene expression of inflammatory and immunoregulatory genes are also upregulated in vitro, and in experimental liver metastasis^26^. Results using this gene signature analysis may indicate that the increased expression of DDR1 gene may also occur in vivo. DDR1 silenced livers developed less metastatic foci than DDR1-expressing ones, which may suggest that depletion of DDR1 in the sinusoids creates a less favorable microenvironment for tumor implantation and colonization. Next, the desmoplastic and angiogenic response generated by the nearby SCs is diminished in DDR1 silenced livers. Thus, it is tempting to speculate that DDR1 phosphorylation and downstream signaling may participate in the generation of microenvironmental conditions for both CRC cell implantation and metastatic foci formation and growth in mice.

Our studies point out HSCs as the SCs with the most abundant DDR1. Furthermore, we find that both freshly isolated, quiescent and tumor-activated HSCs express DDR1. We previously reported that HSCs start to express DDR2 once these cells initiate their activation program^44^. Thus, DDR1 and DDR2 expression patterns differ in HSCs. We and others have previously shown that activated HSCs play a major role as a source of migratory factors for tumor cells, and pro-angiogenic factors for LSECs^32,45^. However, these data should be interpreted with caution as the gene analyses (Table 3) need to be further validated both at the RNA and protein levels. In vitro analysis revealed a role for DDR1 in HSCs expression of genes related to cell migration and secretion of pro-migratory chemotactic factors for LSECs and tumor cells, such as interleukins, CXC chemokines, and MMPs. Chemokines mediate non-inflammatory cell migration and have been implicated in CRC metastasis^30^. These in vitro results may explain the in vivo reduction of angiogenesis and tumor growth in the metastatic tumors of hepatic DDR1-silenced mice. Alternatively, it may also derive from the reduction in the density of activated HSCs inside the tumor in DDR1-silenced livers. In this regard, important genes that regulate cell proliferation and HSCs activation, such as those for the PDGF receptors^46,47^, are less expressed in siDDR1 HSCs, which may further explain the reduced number of activated HSCs in the tumor stroma of hepatic DDR1 silenced mice.

Collagen deposits may act as signaling sources for DDR1 phosphorylation. Hepatic collagen at the sinusoidal areas mainly derives from activated HSCs. In turn, collagen further activates HSCs creating a positive feedback loop. A role for autocrine collagen type I in HSCs DDR1 phosphorylation may be envisioned, as occurred for DDR2^48^. Furthermore, siDDR1 HSCs expressed lower levels of genes for collagen II, III and IV, and for others related to collagen catabolism and MMPs. Such decrement may account in part for the reduced collagen and MMPs activity in the micrometastases of hepatic DDR1-silenced mice. An alternative source of stromal collagen could be the tumor cells. RT-qPCR and Western blot analysis shows that C26 express collagen I (Salado C, personal communication), as human HT29 colon carcinoma cells do^43^.

Remodeling of the collagen matrix aid tumor progression. In the liver, MMPs regulate tissue levels of collagen and participate in the crosstalk between SCs populations, and between the tumor and the SCs^26^. Using a DDR1 kinase inhibitor, we found that expression of MMP2 and MMP9 in response to collagen I was DDR1kinase-dependent in HSCs, and to a lesser extent in KCs. Nevertheless, recent studies reveal a role for DDR1-collagen-independent or DDR1 kinase-independent in cell behavior^3^, such as collective migration in tumor cells^49^, which may explain the observed discrepancies between inhibition of PDDR1 and reduction of MMPs expression.

Our study does not rule out a direct effect of DDR1 silencing in hepatocytes. Hepatocytes promote HSCs recruitment and activation^50^. In our hands, freshly isolated murine hepatocytes express DDR1 (Romayor I, unpublished data). Moreover, Lee et al. have recently unveiled their direct implication in the formation of the pro-metastatic niche^51^. Furthermore, our work utilizes an experimental model of liver metastasis. Whether the same mechanisms occur in the human liver and its relevance in the development of metastatic CRC remain to be assessed. Finally, our studies open the door for future analyses on a role for DDR1 in other liver pathologies where excessive collagen is produced and the contribution of SCs is critical, such as fibrosis and primary liver cancer.

## Material and methods

### Animals

Balb/c male mice (6–8 weeks old) were obtained from Charles River (Barcelona, Spain). Housing, care, and experimental conditions were carried out in conformity with institutional guidelines and national and international laws for experimental animal care. The animals were fed ad libitum with standard chow and water. All the proceedings were approved by the Basque Country University Ethical Committee (CEID) and by national and international guidelines regarding the protection and care of animals used for scientific purposes.

### Isolation of murine hepatic SCs

The isolation of liver SCs, namely HSCs, KCs and LSECs was performed according to the protocol established by Smedsrød and Pertoft^52^ with slight modifications^29,40^. Briefly, the liver was perfused by 0.02% collagenase type IV (Sigma-Aldrich) solution, followed by isopycnic centrifugation on Percoll (MP Biomedicals). HSCs were recollected from the upper phase and LSECs from the lower phase. SCs were assayed for purity prior to culture by Western blot using specific markers for each cell type: GFAP for HSCs, F4/80 for KCs and CD146 for LSECs (see Western Blot section below). KCs were further purified by their high capacity to adhere rapidly to plastic surfaces. HSCs were cultured at 4 × 10^5^ cells/cm^2^, KCs were cultured at 2 × 10^5^ cells/cm^2^ and LSECs were seeded at 8 × 10^5^ cells/cm^2^ onto 1% rat tail collagen I pre-coated plates (Thermo Fisher Scientific). Freshly isolated cells were incubated for 3 h in RPMI-1640 medium with 5% fetal bovine serum (FBS), penicillin, streptomycin, amphotericin B and gentamycin to allow them to recover and attach to the dishes. Then, media was changed to RPMI-1640 medium with 5% FBS, and antibiotics/antimycotics. KCs and LSECs were used right afterward to retain functionality^52,53^. HSCs were incubated for 2 days prior to use to allow full recovery^40^.

### Murine cell lines

C26 colon carcinoma cells, 3T3 skin fibroblasts and J774A.1 macrophages were obtained from the American Type Culture Collection (ATCC, LGC Standards SLU). C26 CRC cell line is syngenic with BALB/c mice. Cell lines were cultured in RPMI-1640 medium with 10% FBS, penicillin, streptomycin and amphotericin B. Cell lines were maintained in sub-confluent cultures and were discarded after ten passages**.**

### In vitro DDR1 phosphorylation activation and blockage

Cells were cultured for 2 h in RPMI-1640 medium containing 10 µg/ml collagen I. In some experiments, cells were pre-treated with DDR1 kinase inhibitor DDR1-IN-1(Tocris Bioscience) for 1 h, then media was changed to one containing 10 µg/ml collagen I and DDR1-IN-1 for 2 h.

### Cell secretomes

C26 Secretomes were obtained as conditioned media (CM) from subconfluent C26 cultures maintained for 24 h in fresh serum-free basal media and antibiotics/antimycotics. Basal SC (Ø) secretomes were obtained from SCs monocultures cultured overnight in fresh basal media plus 1% FBS. Then, media was changed with fresh basal media plus 1% FBS for another 24 h. To obtain tumor-activated (TA) SCs secretomes, SC cells were maintained overnight in the presence of tumor secretomes, diluted 1:2 in fresh basal media plus 1% FBS. Then, media was removed, and new basal media plus 1% FBS were added to the cultures. All CM were collected after 24 h, centrifuged to remove cell debris, and stored at – 20 °C for further use. In some experiments, SCs treated overnight with basal media or with tumor secretomes received or not DDR1-IN-1 for 1 h, then media was changed to fresh basal media containing 10 µg/ml collagen I and DDR1-IN-1 for 2 h. Then, media was changed again to basal media plus 1% FBS, centrifuged and collected 10 h afterwards.

### Cell immunofluorescence

SCs were cultured in coverslips, fixed with 4% paraformaldehyde and incubated in blocking solution (3% BSA in PBS). Next, cells were incubated with rabbit anti-DDR1 (1:200) (Cloud-Clone Corp) overnight at 4 °C and Cy3 goat anti-rabbit (1:2000) fluorescent secondary antibody (Abcam). Actin fibers were stained using Phalloidin-Fluor 488 (1:100). Cells nuclei were stained with Hoechst 33342 (1:1000). Cells were observed using a Zeiss LM800 confocal microscope.

### In vitro HSCs DDR1 silencing

HSCs monocultures maintained in culture for 2 days after isolation were transfected with the DDR1 siRNASTABLE (siDDR1), or non-targeting control siRNASTABLE (siØ) (Dharmacon). Then, media was changed to fresh basal media with 1% FBS, and cells remained untreated for 3 days. DDR1 protein levels were analyzed by Western blot.

### RT-qPCR

SCs monocultures under basal conditions (Ø) were generated by culture for 1 h in fresh basal media supplemented with 5% FBS and antibiotics/antimycotics. SCs monocultures under tumor activated conditions (TA) were generated after 1-h incubation with tumor secretomes, diluted 1:2 in fresh basal media at a 5% FBS final concentration. Next, total RNA was extracted using Norgen FFPE RNA Purification kit (Norgen Biotek Corp). RNA concentration and quality were assessed by NanoDrop spectrophotometer (Thermo Fisher Scientific). 0.5–1 µg RNA was reverse transcribed into cDNA with iScript Reverse Transcription Supermix (BIO-RAD). Quantification of cDNA template was performed using iTaq Universal SYBR Green Supermix (BIO-RAD) in an ABI 7900HT (Life Technologies). PCR primers were: COX2 F, TGCACTATGGTTACAAAAGCTGG and R, TCGGAAGCTCCTTATTTCCCTT; IL6 F, TCTATACCACTTCACAAGTCGGA and R, GAATTGCCATTGCACAACTCTTT; IL10 F, ACAGCCGGGAAGACAATAACT and R, GCAGCTCTAGGAGCATGTGG; PDGF F, TGAGAACCAGCAGAGGCATTT and R, GCCACCATGCTGATTTCCAG; VEGF F, TGTACCTCCACCATGCCAAG and R, ACTTGATCACTTCATGGGACTTCT; COL1A F, TGGGCATCTGGTTTAGCCTT and R, TGACTGTCTTGCCCCAAGTT; DDR1 F, GCGTCTGTCTGCGGGTAGAG and R, ACGGCCTCAGATAAATACATTGTCT; GAPDH (housekeeping) F, GTATGACTCCACTCACGGCAA and R, CTTCCCATTCTCGGCCTTG. Relative expression of target genes was normalized to the internal control gene GAPDH by the ΔΔCt method. Data were generated by the use of specific software (ABI Prism, SDS2.0, Life Technologies) after normalization.

### Western blot

Cells were lysed in Laemmli sample buffer with 1% beta-mercaptoethanol at a ratio of 100 µl/10^6^ cells. Protein quantification was performed using TCA (Fluka Biochemika). Lysates were used in the following amounts: 10 µg (HSCs, KCs, LSECs) or 20 µg (3T3 and J774A.1). Electrophoresis was performed in 15% SDS PAGE gels (to detect GFAP, F4/80, CD146 and CASP3) or 10% SDS PAGE gels (to detect DDR1 and PDDR1). Proteins were transferred onto nitrocellulose membranes. All membranes were cut in two. One part was incubated overnight at 4 °C with one or more of the following antibodies: rabbit anti-GFAP (1:2000, Abcam), rat anti-F4/80 (1:1000, BIO-RAD), rabbit anti-CD146 (1:1000, Abcam), rabbit anti-DDR1, center region (1:1000, GeneTex), anti-DDR1 (1:1000, Cell Signaling Technology), anti-DDR1 (1:1000, gently donated by Regeneron Pharmaceuticals), rabbit anti-PDDR1 (Tyr 513) (1:500, St. John´s Laboratory), rabbit anti-CASP3 (1:1000, Cell Signaling Technology). The other part was incubated overnight at 4 °C with mouse anti-GAPDH (1:1000, BIO-RAD) or rabbit anti-αTubulin (Abcam). Then, membranes were incubated for 1 h with specific secondary antibodies: streptavidin-HRP conjugated (1:500) to detect GFAP, F4/80, CD146 and GAPDH, or Ultra-Sensitive ABC Peroxidase Staining Kit (Thermo Fisher Scientific) for DDR1 and PDDR1 detection. Similar results were obtained using any of the anti-DDR1 antibodies. When adequate, bands were visualized using LuminataTM Crescendo Western HRP Substrate (Millipore). Blots were imaged with SynGene chemiluminescence box. FIJI-ImageJ software was used to quantify protein expression. Protein expression was normalized to GAPDH or αTubulin expression.

### Zymography

Gelatinase/collagenase activity in the culture supernatants was performed as previously described^31^. Loading of samples was standardized based on the amount of total protein. SynGene chemiluminescence box was used to image the gels. FIJI-ImageJ software was utilized to quantify MMPs expression.

### Cell migration assay across collagen I

2 × 10^4^ cells were shed on 0.8 µm-pore diameter inserts (Corning) pre-coated with 1% collagen I. Inserts were placed on top of 2 cm^2^ wells containing SCs secretomes, diluted 1:2 with RPMI-1640 media at 2.5% FBS final concentration. Cells that had migrated to the lower side of the inserts, after 24 h (for C26) or 40 h (for LSECs), were counted in 10 20x-fields per insert, using FIJI-ImageJ software.

### Cell proliferation assay

5 × 10^3^ cells/ml were seeded in 96 wells plates (Sarstedt) and maintained for 3 h in RPMI-1640 FBS-free media. Then, cells were incubated with SCs secretomes diluted 1:2 with culture media at 2.5% FBS final concentration for 48 h. Their proliferation rate was measured using PrestoBlue Cell Viability Reagent (Thermo Fisher Scientific), following the manufacturer’s instructions.

### Gene array

Total RNA from primary HSCs was isolated using RNeasy columns (Qiagen GmbH). Three cell samples were used for each siRNA. Samples quantity and quality were measured with a capillary 2100 Bioanalyzer and mRNA was labeled using the LRILAK PLUS two-color kit (Agilent Technologies). The Cy3-ctp and Cy5-ctp labeled cDNAs were isolated by the RNeasy Mini kit (Qiagen GmbH). These samples were hybridized using Custom Microarray GE, 4 × 44 k according to the manufacturer´s instructions. Slides were scanned using the DNA microarray scanner G2505B (Agilent Technologies) and the images were analyzed with the Agilent Scan Software Feature Extraction v9.5 for normalization and data analysis. Those genes which were 2 fold up-regulated or down-regulated in the hybridization were introduced into the AmiGO 2 Gen ontology database. Bonferroni-corrected p values less than 0.05 were regarded as a statistically significant association. The p-value denotes the significance of GO term enrichment in the differentially expressed gene list.

### In vivo hepatic DDR1 silencing

Mice were injected with 1.5 mg/kg/day with the DDR1 siDDR1, siØ or saline solution through their tail veins for three consecutive days. Hepatic DDR1 protein levels were measured by Western blot 8 days afterwards.

### Experimental hepatic metastases

Metastases were produced in mice after the in vivo hepatic DDR1 silencing. C26 cells were intrasplenically injected with 1.5 × 10^3^ cells/g body weight in syngenic BALB/c mice. After 12 days, mice were killed, and their livers were processed for immunofluorescence analysis. All animals received humane care according to the criteria outlined in the "Guide for the Care and Use of Laboratory Animals" published by the NIH. The experiment was repeated twice (n = 24). Livers were frozen in TissueTek OCT (VWR International) and stored at – 80 °C for further use. Sections of 7 µm obtained at three different tissue levels (1 mm away from each other) per liver were used for hematoxylin/eosin staining and immunofluorescence. Hematoxylin/eosin staining was used to quantify the percentage of liver tissue occupied by the tumor foci (metastatic index) and the number of metastatic foci per liver (density index). Foci were arbitrarily divided into four groups according to their developmental stage and size^6^.

### Tissue immunofluorescence

Liver sections (7 µm thick) were fixed in cold acetone, and stained with hematoxylin/eosin staining. For triple immunohistochemical analyses, sections were reacted with rat anti-mouse CD31 (1:100, Dako), rabbit anti-mouse Desmin (1:200, Dako), and mouse anti-human α-SMA (1:50, Sigma-Aldrich) followed by the appropriate fluorescent secondary antibodies (n = 72). Serial sections were incubated with either rat anti-F4/80 (1:100, BIO-RAD) (n = 36) or mouse anti-Ki67 (1:200; Sigma-Aldrich) (n = 36), followed by appropriate fluorescent secondary antibodies. Irrelevant appropriate immunoglobulins were used as negative controls. Metastases were microphotographed using a Zeiss Axioskop fluorescence microscope under the same conditions. Specific immunostaining was quantified using FIJI-ImageJ software. Results were expressed as the percentage of specifically colored tissue area relative to the whole micrometastatic foci area.

### Picrosirius Red staining

Collagen detection was performed with Picrosirius red staining as previously described^8^.

### In situ zymography

Zymography was performed on cryostat sections using DQ gelatin conjugated with fluorescein (Thermo Fisher Scientific) following the manufacturer´s instructions.

### Statistical analysis

Each experiment was performed three times, except for the in vivo metastasis. Statistical analysis was performed using GraphPad Prism statistical software (version 5.0). Significance was assessed using the Student's two-tailed unpaired t-test and ANOVA test, as appropriate. Data are expressed as the mean ± standard deviation (SD).

## Supplementary information

Supplementary Information

## Data Availability

All data generated or analyzed during this study are included in this published article (and its Supplementary Information files). The datasets generated during and/or analyzed during the current study are available from the corresponding author on reasonable request.

## Abbreviations

DDR1: Discoidin domain receptor 1
PDDR1: PhosphoDDR1
ECM: Extracellular matrix
SCs: Sinusoidal cells
HSC: Hepatic stellate cell
KC: Kupffer cell
LSEC: Liver sinusoidal endothelial cell
CRC: Colorectal carcinoma
α-SMA: Alpha-smooth muscle actin
MMP: Matrix metalloproteinase
