# Supplementary material for: Silencing of sinusoidal DDR1 reduces murine liver metastasis by colon carcinoma
Source: Sci Rep. 2020 Oct 27;10:18398. doi: 10.1038/s41598-020-75395-w (PMC7591579; doi:10.1038/s41598-020-75395-w)
Supplement: Supplementary file 1 — Supplementary Information [file 41598_2020_75395_MOESM1_ESM.pdf]

**Silencing of sinusoidal DDR1 reduces murine liver metastasis by colon carcinoma.**

**Irene Romayor, Iker Badiola, Aitor Benedicto, Joana Márquez, Alba Herrero, Beatriz Arteta, and Elvira Olaso**

## Supplementary Figure S1

RT-PCR analysis on selected genes in SCs under basal and tumor-activated conditions

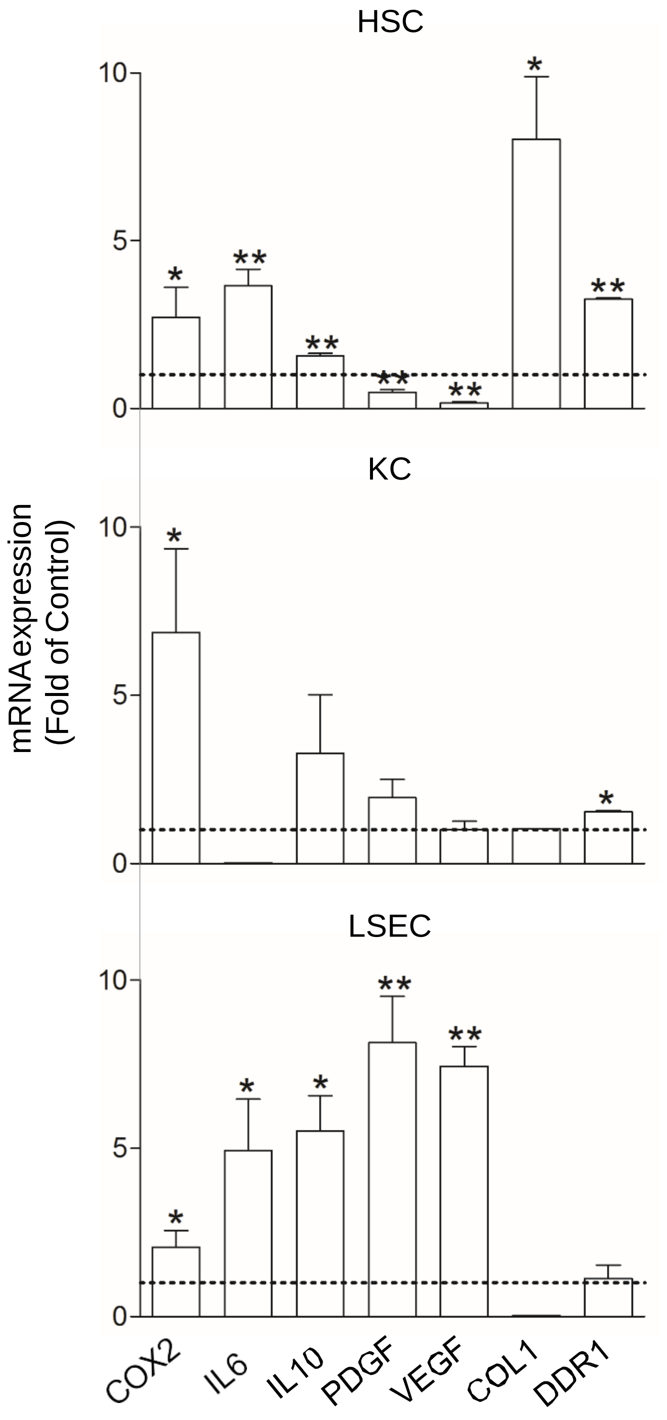

**Silencing of sinusoidal DDR1 reduces murine liver metastasis by colon carcinoma.**

**Irene Romayor, Iker Badiola, Aitor Benedicto, Joana Márquez, Alba Herrero, Beatriz Arteta, and Elvira Olaso**

## Supplementary Figure S2

DDR1 expression in human LX2 HSCs and in DDR1+/+ and DDR1-/- mouse skin fibroblasts

**a**

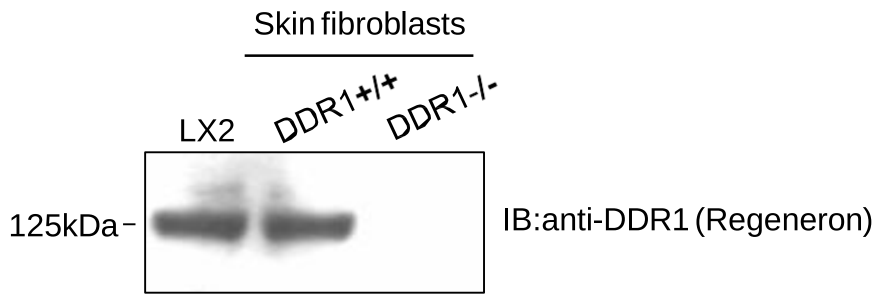


**b**

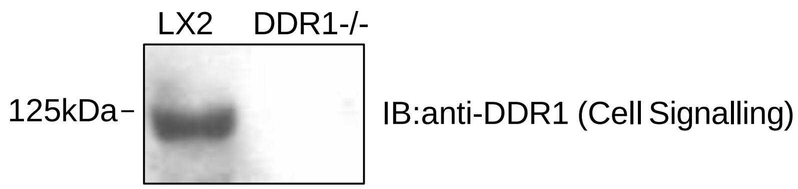

**Silencing of sinusoidal DDR1 reduces murine liver metastasis by colon carcinoma.**

**Irene Romayor, Iker Badiola, Aitor Benedicto, Joana Márquez, Alba Herrero, Beatriz Arteta, and Elvira Olaso**

**Supplementary Figure S3**

DDR1 expression and phosphorylation in 3T3 and J774A.1 cells

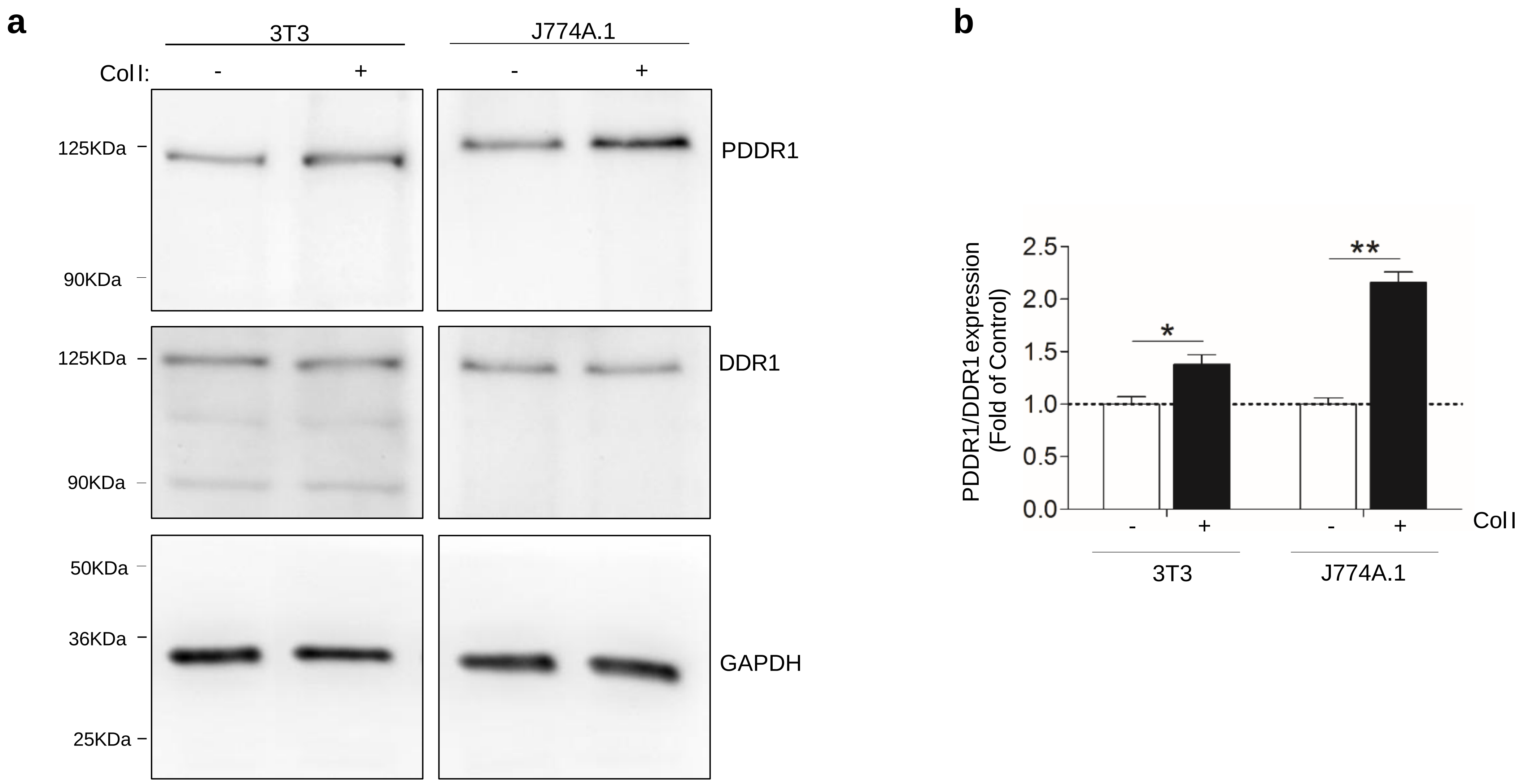

# Silencing of sinusoidal DDR1 reduces murine liver metastasis by colon carcinoma.

Irene Romayor, Iker Badiola, Aitor Benedicto, Joana Márquez, Alba Herrero, Beatriz Arteta, and Elvira Olaso

## Supplementary Figure S4

DDR1 expression and phosphorylation in 3T3 in response to DDR1-IN-1

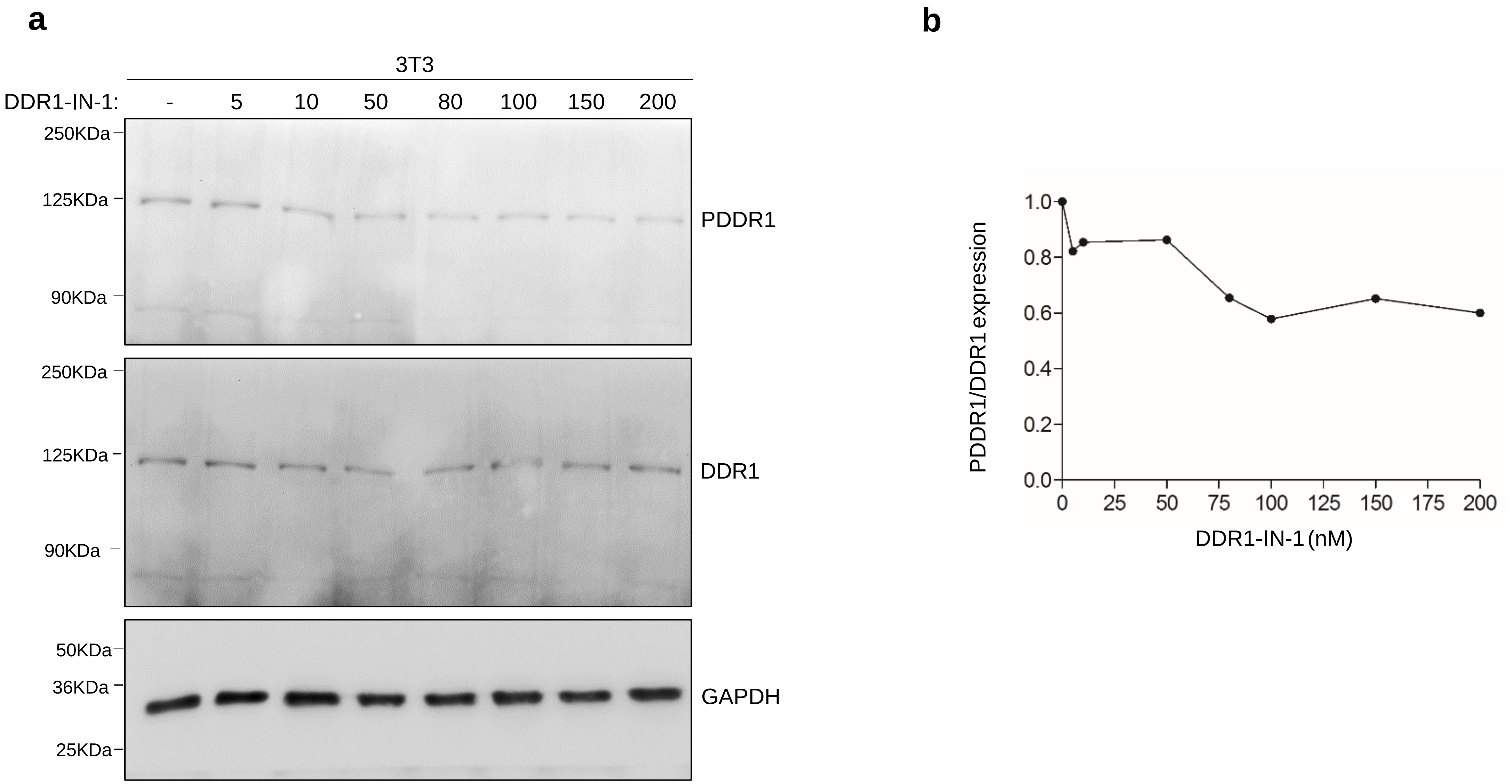

**Silencing of sinusoidal DDR1 reduces murine liver metastasis by colon carcinoma.**

**Irene Romayor, Iker Badiola, Aitor Benedicto, Joana Márquez, Alba Herrero, Beatriz Arteta, and Elvira Olaso**

## Supplementary Figure S5

DDR1 expression and phosphorylation in J774A.1 cells in response to DDR1-IN-1

**a**

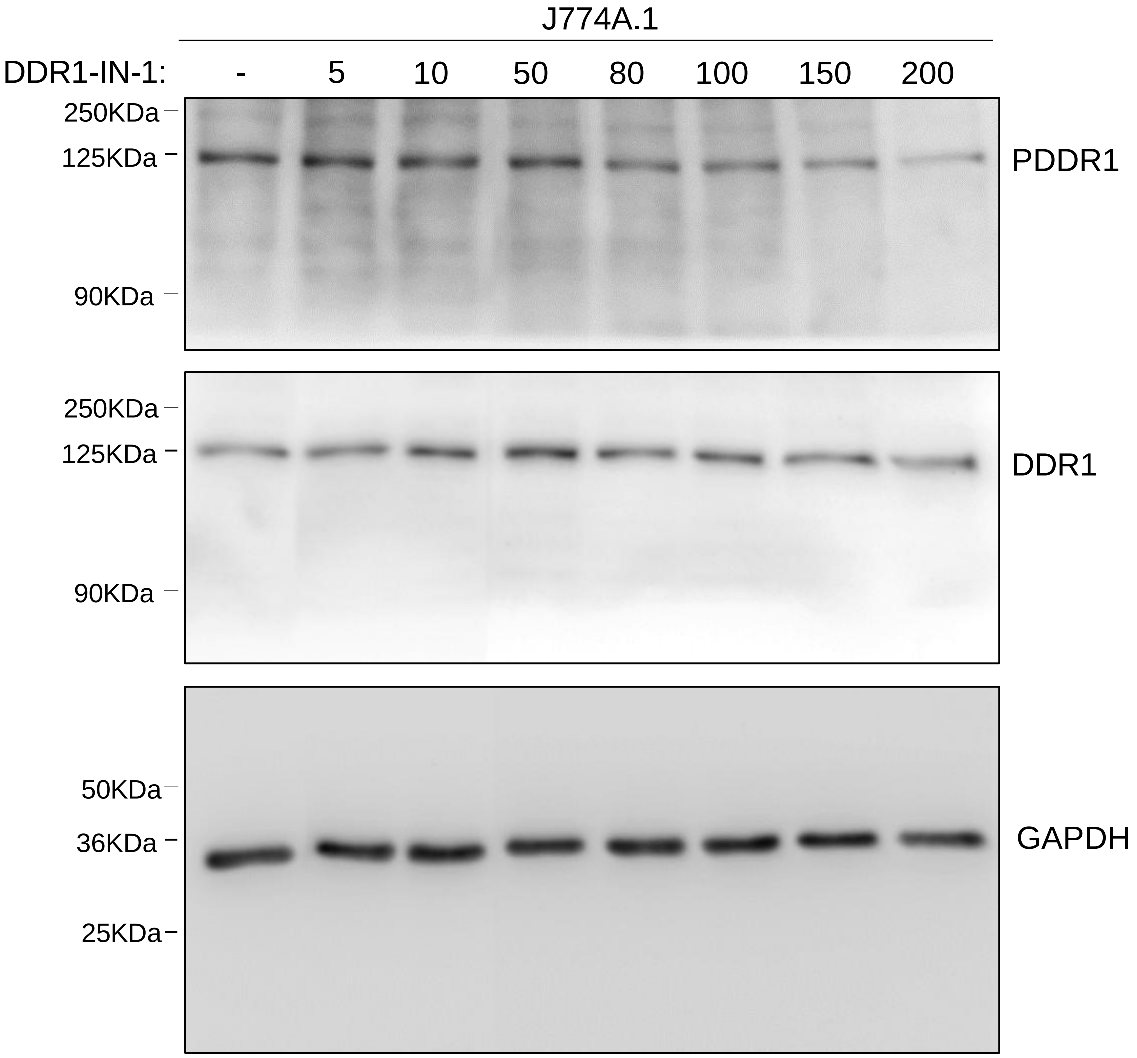


**b**

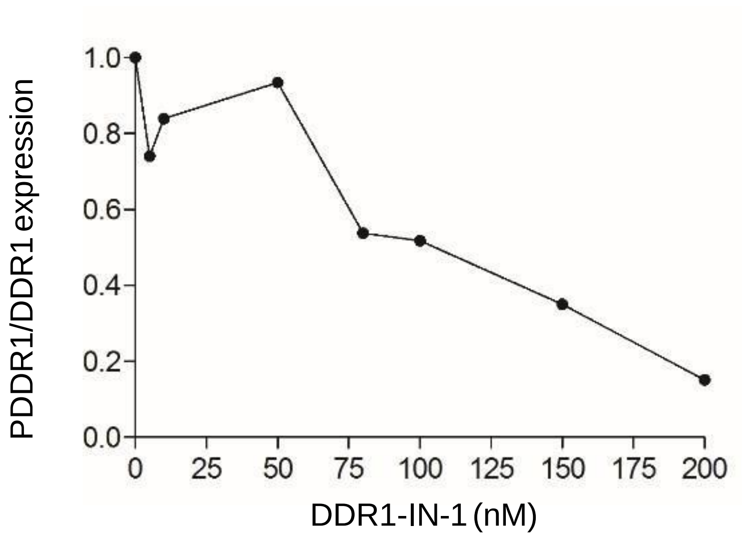

**Silencing of sinusoidal DDR1 reduces murine liver metastasis by colon carcinoma.**

**Irene Romayor, Iker Badiola, Aitor Benedicto, Joana Márquez, Alba Herrero, Beatriz Arteta, and Elvira Olaso**

## Supplementary Figure S6

Caspase 3 expression in SCs in response to DDR1-IN-1

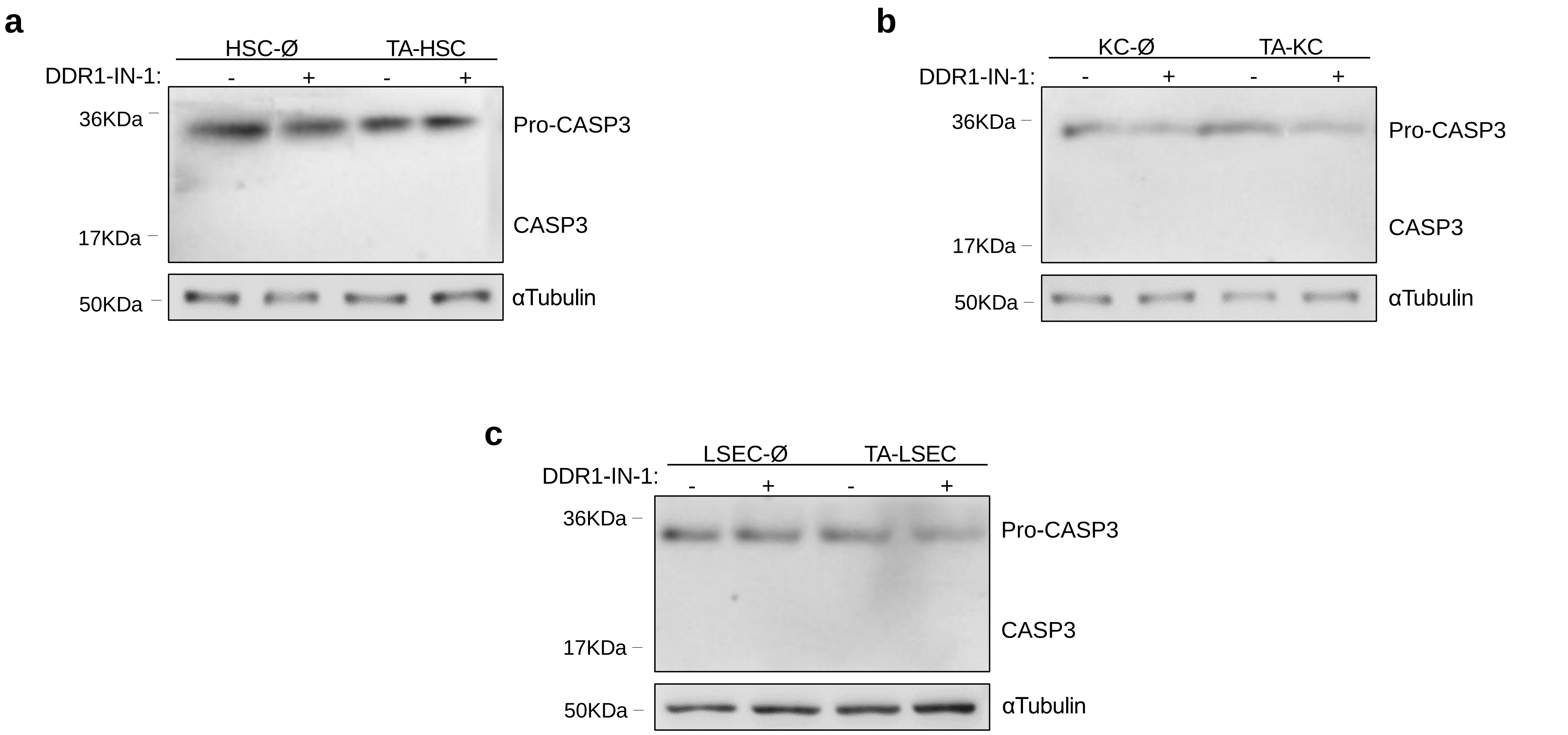

**Silencing of sinusoidal DDR1 reduces murine liver metastasis by colon carcinoma.**

**Irene Romayor, Iker Badiola, Aitor Benedicto, Joana Márquez, Alba Herrero, Beatriz Arteta, and Elvira Olaso**

## Supplementary Figure S7

Active MMP9 expression in KCs in response to DDR1-IN-1 and C26 cells secretome

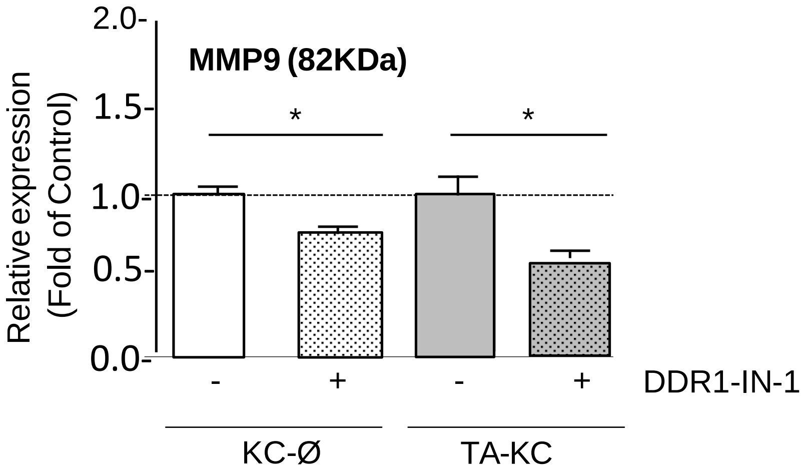

**Silencing of sinusoidal DDR1 reduces murine liver metastasis by colon carcinoma.**

**Irene Romayor, Iker Badiola, Aitor Benedicto, Joana Márquez, Alba Herrero, Beatriz Arteta, and Elvira Olaso**

## Supplementary Figure S8

Full-length gels and blots

**Figure 1a**

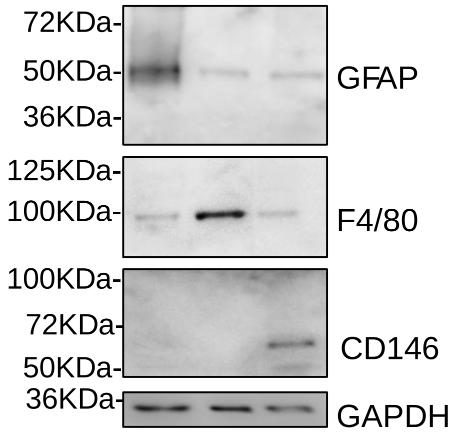


**Figure 1b**

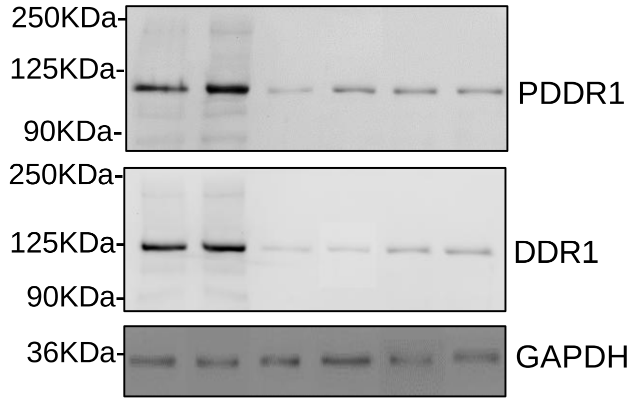


**Figure 2a**

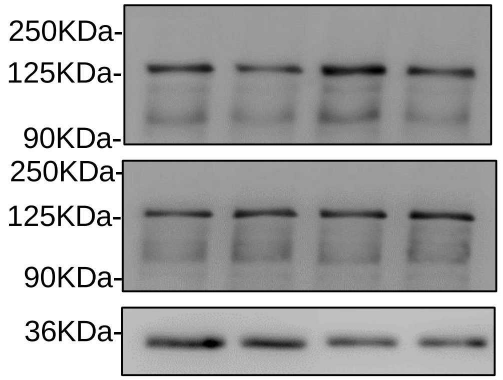


**Figure 2b**

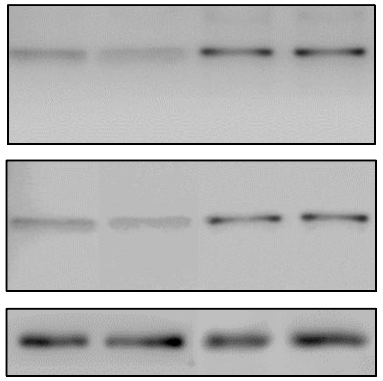

**Figure 2c**

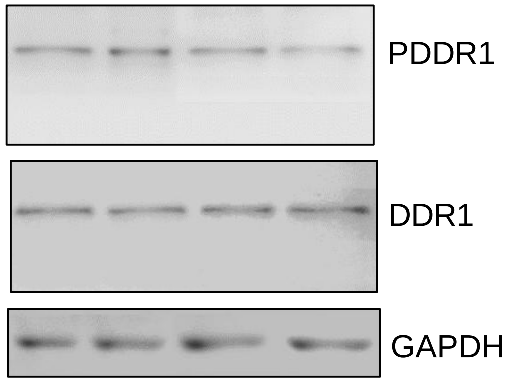


**Figure 5a**

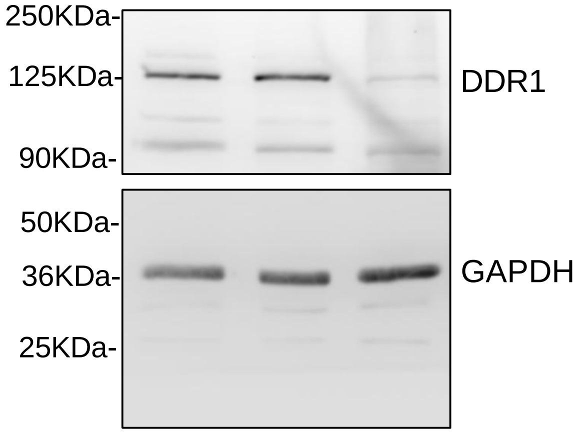


**Figure 6b**

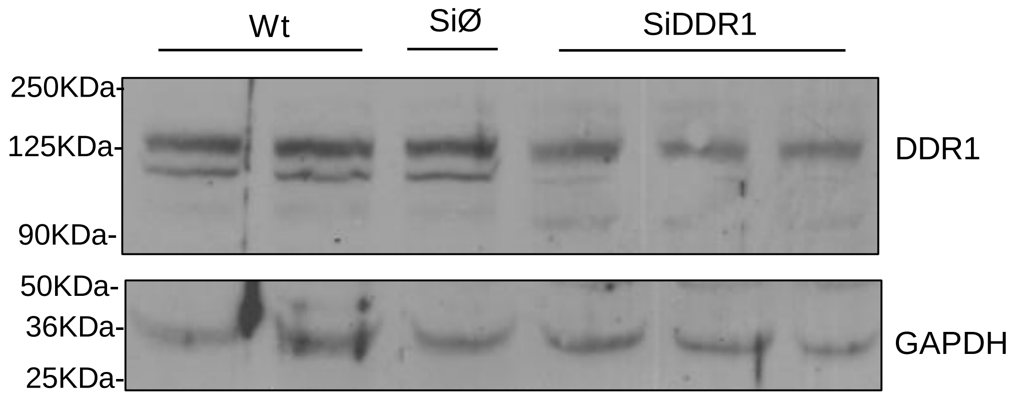

# Silencing of sinusoidal DDR1 reduces murine liver metastasis by colon carcinoma.

Irene Romayor, Iker Badiola, Aitor Benedicto, Joana Márquez, Alba Herrero, Beatriz Arteta, and Elvira Olaso

## SUPPLEMENTARY FIGURE LEGENDS

Supplementary Figure S1. Increased DDR1 in SCs by C26 cells secretomes. Freshly isolated mouse SCs as monocultures of HSCs, KCs and LSECs were cultured in the presence of C26 cells secretome or in basal media for 1 hour. Next, RT-qPCR was performed to analyze gene expression of DDR1 in correlation with genes that have previously shown to be altered within the SCs that conform the hepatic tumor microenvironment. Such gene profile includes genes related to inflammation (COX2, IL6), immunosuppression (IL10), angiogenesis (PDGF, VEGF) and COL1. Results for each gene was correlated with that of housekeeping GAPDH. Next, relative expression of each gene in every SCs monoculture pretreated with C26 secretomes was divided by the relative expression of that gene in the correspondent SCs monoculture maintained under basal conditions. Data are expressed as the means ± SD. $^{*}P < 0.1$, $^{**}P < 0.01$. Each experiment was repeated three times with fresh isolated SCs and a freshly obtained C26 secretome.

Supplementary Figure S2. DDR1 expression in human HSCs LX2 and skin fibroblasts. (a, b): Western blot of total DDR1 expression in LX2 and skin fibroblasts cell lines derived from DDR1+/+ or from DDR1-/- mice. Two different antibodies were used.

Supplementary Figure S3. DDR1 phosphorylation by collagen type I in 3T3 fibroblasts and J774A.1 macrophages. 3T3 and J774A.1 cell lines were treated with collagen I (a): Western blot of PDDR1 and total DDR1 expression in the cell lines in response to collagen I. (b): Histogram on the average data from three independent experiments. Data are expressed as means ± SD. $^{*}P < 0.1$, $^{**}P < 0.01$.

Supplementary Figure S4. Blockage of DDR1 phosphorylation by DDR1-IN-1 in 3T3 fibroblasts. 3T3 fibroblasts were treated with collagen I and DDR1-IN-1 (a): Western blot of PDDR1 and total DDR1 expression in 3T3 fibroblasts cultured with increasing concentrations of DDR1-IN-1 (0-200nM). (b): Histogram on the average data from one experiment.

Supplementary Figure S5. Blockage of DDR1 phosphorylation by DDR1-IN-1 in J774A.1 macrophages. J774A.1 macrophages were treated with collagen I and DDR1-IN-1 (a): Western blot of PDDR1 and total DDR1 expression in J774A.1 cultured with increasing concentrations of DDR1-IN-1 (0-200nM). (b): Histogram on the average data from one experiment.

Supplementary Figure S6. Apoptotic rate of liver SCs after DDR1-IN-1 treatment. Freshly isolated SCs as monocultures of control (Ø) and tumor activated (TA) HSCs, KCs and LSECs were cultured in the presence of collagen I and DDR1-IN-1. (a-c): Western blot of Pro-CASP3 and CASP3 expression in HSCs (a), KCs (b) and LSECs (c) in response to 70nM DDR1-IN-1. Each experiment was repeated three times with fresh isolated SCs and a freshly obtained C26 secretome.

Supplementary Figure S7. DDR1 kinase−dependent expression of active MMP9 by KCs. Histogram based on the results shown in Figure 3. In brief, primary monocultures of basal (Ø) or tumor-activated (TA) KCs were pretreated or not with DDR1-IN-1 and collagen I. Then media was changed to fresh basal media, and secretomes collected ten hours afterwards, and submitted to gelatin zymography. The histogram represents a semi-quantitation of the three experiment performed. Data represents the amount of MMP9 in KCs secretomes. Data are expressed as the means ± SD. $^{*}P < 0.01$.

Supplementary Figure S8. Full-length blots. Full western blots from Figures 1, 2, 5 and 6.
